# Recruitment Traits Could Influence Species’ Geographical Range: A Case Study in the Genus *Saxifraga* L.

**DOI:** 10.3389/fpls.2022.827330

**Published:** 2022-05-13

**Authors:** Vera Margreiter, Francesco Porro, Andrea Mondoni, Brigitta Erschbamer

**Affiliations:** ^1^Department of Botany, University of Innsbruck, Innsbruck, Austria; ^2^Department of Earth and Environmental Science, University of Pavia, Pavia, Italy

**Keywords:** common garden, phenology, regeneration niche, seed sowing experiment, intraspecific variation, germination

## Abstract

The reasons why some species occur widespread, while related species have restricted geographical ranges have been attributed to habitat specialization or ecological niche breadth. For species in the genus *Saxifraga*, habitat specialization alone cannot explain the distributional differences observed. We hypothesize that recruitment traits (i.e., germination, emergence, and survival) may account for differences in geographical ranges and that early life stages correlate to survival. We studied recruitment responses in 13 widespread and 12 narrow-ranged *Saxifraga* species in the laboratory and common garden experiments using seeds collected from 79 populations in the European Alps. We found that in the laboratory cold temperature led to higher germination percentages compared with warm temperature for both distribution groups. This represents an exception to the general assumption that alpine species require warm cues for germination. In warm laboratory temperatures, widespread species germinated better than narrow-ranged species, indicating a greater tolerance of warm temperatures for the former. Subsequent to germination, recruitment traits between the two distribution groups were lower or null in the common garden, suggesting that the impact of recruitment on species’ geographical ranges occurs at the earliest life stage. Mean time to emergence of narrow-ranged species showed lower variability than that of widespread species. Consistently, intraspecific variation of mean annual temperatures between seed collection sites was lower for narrow-ranged species, indicating a close relationship between home sites and emergence time. Emergence percentage was a strong predictor of survival only for widespread species, underlining that seed and seedling functional traits differ between distribution groups, which require further research. Our results support the view that early life stages are critical to population dynamics and thus can influence species’ geographical ranges. The wider responses to climatic conditions in widespread species may have facilitated their spread across the Alps. Our results also suggest that all *Saxifraga* species face a considerable threat from climate warming due to their overall cold-adapted recruitment niche.

## Introduction

The reasons why some species occur widespread (e.g., in a variety of habitats or stretched over a wide geographical range) and some closely related species occupy a narrow geographical range (hereafter referred to as narrow-ranged) has been of interest to biologists for decades. Current studies investigated the status of species’ rarity (e.g., Rabinowitz, 1982; Broennimann et al., 2005), ecophysiological traits comparing widespread and narrow-ranged species (e.g., Lavergne et al., 2004; Mouillot et al., 2013), habitat preferences of endemic species (Essl et al., 2009), and loss of rare species due to land-use practices globally (e.g., Newbold et al., 2018). However, no sufficiently comprehensive explanation of geographical distribution patterns has been found until the present. In this context, little attention has been given to the role of seedling recruitment (e.g., Münzbergová, 2004; Larson and Funk, 2016; Tudela-Isanta et al., 2018). Seedling recruitment includes key demographic processes such as seed germination (the transition from a quiescent or dormant seed to a germinated seed), emergence (the transition from a germinated seed to an emerged seedling), and seedling survival (the persistence of established seedlings through the first growing season; Fenner and Thompson, 2005). In temperate areas, seedling survival during the first winter period is additionally crucial for seedling recruitment (Niederfriniger Schlag and Erschbamer, 2000; Mondoni et al., 2015, 2020; Margreiter et al., 2021).

Each transition phase plays an important role in population dynamics (Donohue et al., 2010; Fraaije et al., 2015; Jiménez-Alfaro et al., 2016; Rosbakh et al., 2020). The influence of one phase on the subsequent one depends on a set of abiotic (e.g., light, soil moisture, temperature, chemicals, and nutrients) and biotic filters (e.g., fungal pathogens, herbivores, competitors, and facilitators), and interactions among these (see Larson and Funk, 2016), making it hard to disentangle single-trait influences on recruitment. In addition, local adaptation has been noted to impact seedling emergence and survival outcomes (Giménez-Benavides et al., 2007). However, by looking at each life phase separately, the individual contribution to possible distributional differences between widespread and narrow-ranged species may be determined since the transition phases are ultimately interlinked. For example, by using data from 37 dry grassland species and partial regression coefficient calculations, Larson et al. (2015) clearly showed that germination and emergence were useful traits to predict seedling survival.

Early life stages may influence species distribution ranges (Donohue et al., 2010; Larson and Funk, 2016), determined by trait niche breadths (Finch et al., 2018). A general hypothesis states that the environmental niche (i.e., the breadth of any trait) is broad when a heterogeneous environment is inhabited, while a homogeneous environment favors narrow niches (Kassen, 2002; Finch et al., 2018). For example, narrow germination niches were linked to small geographic ranges in geophytes, hemicryptophytes, and shrubs (Ascough et al., 2007; Ranieri et al., 2012), while inhabiting a broader geographic and climatic range was found to be correlated with broad germination niches in hemicryptophytes and chamaephytes (Brändle et al., 2003; Luna and Moreno, 2010). Conversely, germination niches varied among congeneric species of *Asclepias* with similar distribution ranges. A narrow germination niche can be caused by high dormancy cues and slow and low germination that might ensure the survival of seedlings by only germinating when conditions are met (Donohue et al., 2010; Rosbakh et al., 2020). Seed dormancy, operating as a bet-hedging strategy, may prevent germination at the first possible opportunity to spread the risk of seedling mortality over a season or year (Ooi et al., 2009; Donohue et al., 2010; Cotado et al., 2020). On the other hand, when germination thrives under a broad set of conditions with low dormancy cues, seedlings may be occasionally exposed to unfavorable post-germination conditions (Mondoni et al., 2015), but may result in overall higher germination outcomes. On expanding these ideas from the germination niche to the regeneration niche (Grubb, 1977), a narrow niche for early life stages may lead to a limited distribution range of adult species, while in contrast, when early life stages have a wider niche, adults may be able to occupy a wider distribution range.

Species-specific characteristics with regard to germination, emergence, and survival have been widely described (e.g., Münzbergová, 2004; Milla et al., 2009; Fraaije et al., 2015; Mondoni et al., 2015, 2020; Larson and Funk, 2016; Walter et al., 2020; Margreiter et al., 2021), which are expressed as interspecific variations (Albert et al., 2010a,b; Fraaije et al., 2015). On the other hand, intraspecific variability was recognized as a key characteristic in ecology (Des Roches et al., 2018) that acts on different organizational levels (Violle et al., 2012; Chen and Giladi, 2020), meaning that variation is important for functional diversity at species, community, and ecosystem levels (Albert et al., 2010a,b; Larson and Funk, 2016; Des Roches et al., 2018). To occupy a niche within a habitat, a certain degree of intraspecific variability is necessary to respond to conditions of that niche at each life-history stage. High intraspecific variation may allow species to perform similarly under different conditions, thereby reflecting a broad niche; on the other hand, when species lack intraspecific variation, performance is only possible under specific conditions (Finch et al., 2018). Intraspecific variation in seed traits is assumed to increase with increasing species’ range size (Luna and Moreno, 2010). However, a screening of recruitment traits of phylogenetically related species has hardly been performed (e.g., Rosbakh et al., 2020; Veselá et al., 2020), and, most importantly, only a few studies covered all transition phases of recruitment, i.e., from germination to seedling survival (see Vázquez-Ramírez and Venn, 2021).

Intraspecific variation of recruitment responses may be especially important in times of an anthropogenic climate change when quick reactions to the environment are essential for species’ persistence (Cochrane et al., 2015; Henn et al., 2018; Walter et al., 2020). For example, populations of a single species may be locally adapted to lower levels of soil moisture, and can therefore germinate in years with lower precipitation compared with populations that remain dormant in the seed bank in the same year (Ooi et al., 2009). On the other hand, rapid climate changes have the potential to disrupt existing environmental cues for both seed germination (Donohue et al., 2005; Walck et al., 2011) and seedling survival (Anderson and Wadgymar, 2020). This risk could be especially high for narrow-ranged species as intraspecific variation may be low because fewer populations exist and restricted gene flow may have lowered fitness. Warming (i.e., temperature increase) has been reported to favor seedling emergence for widespread alpine species (Mondoni et al., 2015, 2020; Vázquez-Ramírez and Venn, 2021), but also disfavoring results were obtained due to biotic interactions (Meineri et al., 2013; Margreiter et al., 2021) and local adaptations (Giménez-Benavides et al., 2007).

The European Alps are home to about 60 *Saxifraga* species (spp.), including a high number of subalpine and alpine species (i.e., species at and above the treeline) and a lower number from the montane zone (Aeschimann et al., 2004). *Saxifraga* spp. colonized Europe in the late Eocene and rapidly diversified after reaching the continent from Northeast Asia (Zhang, 2013; Ebersbach et al., 2017). Thus, the species occurring in Europe are viewed as being considerably old and their distributional ranges were influenced by habitat specialization (Hegi, 1923). Species that are rare or endemic in the Alps may therefore be described as “old rare species,” meaning that they show a natural patchy distribution as a result of habitat preferences (Meier and Holderegger, 1998). However, for narrow-ranged species in this study, there are suitable habitats not occupied outside the isolated populations, suggesting that the narrow range cannot be explained by habitat specificity alone. For example, *S. squarrosa* grows on calcareous bedrock in rock crevices of the Southeast Alps, and although calcareous bedrock and similar habitats are also abundant in the Northern and Northeastern Calcareous Alps, *S. squarrosa* is not found there. The widespread species *S. caesia*, on the other hand, occurs in the entire calcareous Alps. The fact that all *Saxifraga* spp. have similarly tiny and light seeds that are easily dispersed by wind makes distribution limitation by dispersal unlikely for *S. squarrosa*. Instead, it presses the question of whether distributional differences between narrow-ranged and widespread *Saxifraga* spp. arise due to differences in their recruitment responses. Moreover, investigations on a similar set of *Saxifraga* spp. suggest that the phylogenetic influence on germination niche, expressed by Pagel’s lambda (Pagel, 1999), is only low to moderate (Porro et al., submitted; λ < 0.55 for seed germination in response to temperature and cold stratification, and their interactions with Landolt ecological indicator values). Thus, the *Saxifraga* genus represents an ideal model for the study of the distribution range, intraspecific variations and their drivers.

The general objective of this study is to assess whether traits of the early life stages of *Saxifraga* spp. can explain the species distribution of this genus in the Alps. Seeds of 25 *Saxifraga* spp. from 79 populations were collected and used in the laboratory and common garden experiments. As each transition phase can restrict recruitment outcomes, each phase was studied separately. We tested the hypotheses that (1) compared with narrow-ranged *Saxifraga* spp., widespread species are characterized by (a) higher levels of recruitment traits [germination, emergence, and survival (both during the first growing season and overwinter)] and (b) greater intraspecific variations in germination and emergence and that (2) early transition stages (i.e., germination and emergence) correlate to survival.

## Materials and Methods

### Study Species and Seed Collection

**FIGURE 1:**
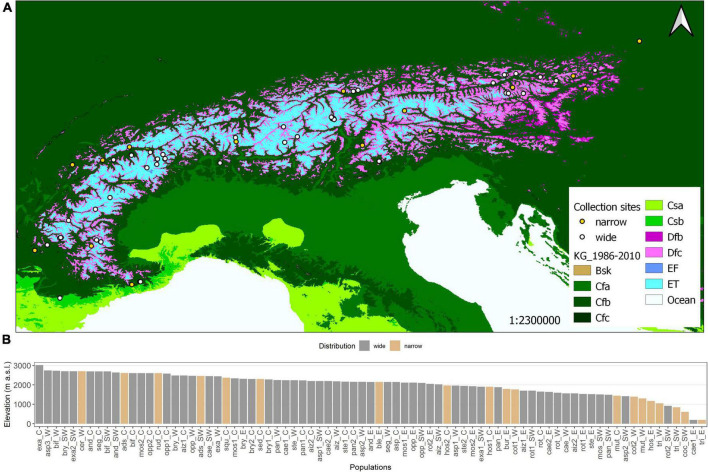
**(A)** Köppen-Geiger climate map (open source) of the European Alps and seed collection sites (points). In total, 79 populations of *Saxifraga* spp. were collected from Southwest (France) to the East (Austria). As some points overlap in the overview, detailed location maps are provided in Supplementary Figure 1. The climatic zones can be classified as arid (Bsk), warm temperate (Cfa, Cfb, Cfc. Csa, Csb), boreal (Dfb, Dfc), and alpine (ET, EF). **(B)** Histogram of elevations of seed collection sites per population, in descending order of elevation.

In total, 25 *Saxifraga* spp. (Table 1) from 79 populations (Figure 1 and Supplementary Table 1) were collected. Life forms (Landolt et al., 2010) include therophytes, hemicryptophytes, and herbaceous chamaephytes (Table 1). Nomenclature refers to Aeschimann et al. (2004); however, two explanations on species shall be made. First, according to phylogenetic studies of Zhang (2013) and Ebersbach et al. (2017), *S. stellaris* may belong to the section/genus *Micranthes*, but we treated *S. stellaris* as part of *Saxifraga*. Second, S. *blepharophylla* is listed as a subspecies of *S. oppositifolia*, but Holderegger and Abbott (2003) declared it as a separate species after applying chloroplast DNA analyses, and so we treated *S. blepharophylla* as a species. Of the 25 species, 13 were classified as widespread and 12 as narrow-ranged species, based on an ‘‘IUCN Species Area of Occupancy’’ (AOO) approach, where AOO is defined as the smallest area of occupancy of a species. To this end, we determined the AOO of each species within the geographic region of the European Alps via the open-source tool GeoCAT,^1^ with data points taken from the Global Biodiversity Information Facility (GBIF). If AOO was smaller than 1,000 km^2^, species were classified as narrow-ranged and > 1,000 km^2^ as widespread (“wide” and “narrow” in Figures and Table 1).

**TABLE 1:** Study species of the genus *Saxifraga* (Saxifragaceae) having a wide or narrow distribution range (decending order by AOO), and number of populations (No. Pop) collected in the European Alps.

*Saxifraga* spp.	No. Pop	AOO Alps (km^2^)	Distribution	Elevations of collection	Lab test (No. Pop)	Common Garden	Seed mass (mg)	T°	K°	L°	F°	W°	R°	N°	H°	D°	LF°	KS°
*bryoides*	5	1,948	wide	2,300–2,703	x	x	0.053	1	3	5	3.5	1	2	1	1	5	c	cs
*rotundifolia*	6	1,840	wide	918–2,048	x	x	0.039	2.5	2	2	4	3	3	4	3	1	h	cs
*paniculata*	5	1,784	wide	1,487–2,244	x	x	0.04	2	3	5	2	1	4	2	1	5	c	ss
*caesia*	6	1,572	wide	196, 1,564–2,449	x	x	0.029	1.5	4	5	2	1	5	1	3	5	c	ss
*aspera*	6	1,568	wide	1,415–2,740	x	x	0.016	2	3	5	2.5	1	2	2	1	5	c	cs
*stellaris*	4	1,504	wide	1,520–2,235	x (3)	x	0.04	2	2	5	4	3	3	2	3	1	c	cs
*androsacea*	3	1,368	wide	2,150–2,698	x	x	0.036	1	2	4	3.5	1	4	2	3	3	c	ss
*seguieri*	2	1,268	wide	2,150, 2,698	x	x	0.036	1	2	5	4	1	2	2	3	3	c	cs
*moschata*	5	1,220	wide	1,503–2,601	x	x	0.038	1	4	5	3	1	4	1	3	5	c	ss
*exarata*	4	1,204	wide	1,900–3,020	x	x	0.053	1	4	5	3	1	2	1	3	5	c	ss
*aizoides*	6	1,140	wide	1,560–2,479	x (5)	x	0.067	2	2	4	4	3	4	2	1	1	c	cs
*biflora*	3	1,140	wide	2,601–2,730	x	x	0.117	1	2	5	3.5	1	4	2	1	5	c	cs
*oppositifolia*	5	1,116	wide	2,096–2,601	x	x	0.118	1	4	5	3.5	3	4	2	1	5	c	cs
*rudolphiana*	1	528	narrow	2,601	x	x	0.07	1	2	5	3.5	3	4	2	1	5	c	cs
*cotyledon*	2	468	narrow	1,390, 1,762	x	x	0.035	2.5	3	4	3	1	2	2	1	5	c	ss
*mutata*	2	468	narrow	1,300, 1,435	x	x	0.036	2.5	2	3	4	3	4	2	1	1	h	ss
*tridactylites*	3	452	narrow	196, 846, 1,050	x	x	0.012	4.5	4	4	2	1	4	2	1	5	t	rs
*hostii*	3	304	narrow	1,900–1,969	x	x	0.048	2	3	5	2	1	5	2	1	5	c	ss
*sedoides*	1	296	narrow	2,300	x	x	0.077	1.5	2	5	3.5	1	5	2	3	5	h	cs
*squarrosa*	1	288	narrow	2,365	x	x	0.029	2	4	5	2	1	5	1	1	5	c	ss
*adscendens*	2	244	narrow	2,458–2,617	x	x	0.011	1.5	4	4	3.5	1	4	4	3	3	t	rs
*blepharophylla*	1	208	narrow	2,150	n.a.	x (2 pots)	n.a.	1	3	5	3.5	1	2	2	1	5	c	cs
*cochlearis*	1	168	narrow	610	x	x	0.05	3	4	4	2	1	5	2	1	5	c	ss
*cernua*	1	28	narrow	2,700	n.a.	x	0.214^#^	1.5	2	4	3.5	1	4	4	3	5	h	ss
*burseriana*	1	n.a.	narrow	1,788	x	x	0.052	2	4	5	1.5	1	5	1	1	5	c	ss

*The distribution range was classified by the area of occupancy (AOO), determined using the GeoCAT tool with data points from the Global Biodiversity Information Facility (GBIF) for the European Alps. Elevations of seed collection (see details for each population in Supplementary Table 1). Columns six and seven list the laboratory test or the common garden experiment and the number of populations in parenthesis in case of diverging from the text. Seed mass (mg/seed) and ecological indicator values per species are listed in the last 12 columns. Landolt et al. (2010) ecological indicator values. T, Temperature value (1–4.5; alpine-nival to warm-colline); K, continentality (1–5; oceanic to continental); L, Light requirement (1–5; strong shade < 3% light to full light); F, soil moisture (1–5; dry to flooded); W, alternating soil moisture (1–3; low variability to non-variable moisture); R, soil reaction (1–5; pH 2.5 to > 6.5); N, Nutrients (1–5; nutrient poor to nutrient rich); H, Humus (1–5; no/little humus to high humus content); D, Aeration of soil (1–3; poor aeration/wet soil to good aeration/sandy soil); LF, Life form (t, therophyte; c, herbaceous chamaeophyte; h, hemicryptophyte); KS, simplified Strategy types (rs, ruderal stress tolerant; cs, competitive stress tolerant; ss, stress tolerant).*

*^#^Breman et al. (2019); n.a., not available.*

Seeds were harvested at the time of natural seed maturity, i.e., Jun–Oct 2016 and 2017. Collections were done across the whole European Alpine arc at elevations from 400 to 3,020 m a.s.l (Figure 1). In addition, seeds from two populations were received from the Botanical Garden in Vienna (S. *caesia*, S. *tridactylites*) situated at 196 m a.s.l. Every population was harvested from at least five individuals. Seed collection was done as a joint effort of the “Alpine Seed Conservation and Research Network” (Müller et al., 2017),^2^ collecting seeds in their countries and sending them to Innsbruck, Austria. The seeds were then cleaned and stored at room temperature (∼20°C, ∼60% relative humidity, up to 6 months) until further processing, i.e., preparing seeds in paper bags for the garden experiment and placing the seeds on Petri dishes for laboratory germination tests. Seed storage in this approach is considered to have a negligible effect on germination potential, as the seeds have subsequently undergone a stratification period to release seed dormancy.

### Laboratory Experiment: Germination

Prior to germination tests, seeds were cold-wet stratified on moist filter paper for 3 months (∼4°C refrigerator). Petri dishes were wrapped in aluminum foil to guarantee darkness. This stratification served to simulate a winter period and break a possible seed dormancy (Schwienbacher et al., 2011; Tudela-Isanta et al., 2018). Petri dishes were checked twice under green-light conditions (to avoid a light impulse) for watering and to replace filter papers in case of fungal infections. After stratification, the seeds were transferred to growth chambers (SANYO MLR-350H, Sanyo Electric Biomedical Co., Ltd., Japan) with two alternating temperature conditions: warm at 25/15°C and cold at 15/5°C. Growth chambers were set to 16 h light (20,000 lx) and 8 h dark (0 lx) with 60% air humidity. The maximum photosynthetic photon flux density was 180 μmol m^–2^s^–1^. These settings were chosen due to the previous knowledge on warm cues for the germination of alpine species (Schwienbacher et al., 2011; Walder and Erschbamer, 2015; Fernández-Pascual et al., 2020) and previous studies showing good germination results with 15/5°C in other *Saxifraga* spp. (e.g., Giménez-Benavides and Milla, 2013).

Ideally, for each species and population, four Petri dishes with 25 seeds per Petri dish were prepared on three layers of filter paper (Ø 90 mm) and moistened with deionized water. Depending on seed availability, the number of seeds placed per Petri dish was adjusted; exact numbers of seeds sown and germination per population are given in Supplementary Table 2. A germination test period lasted 50 days. The Petri dishes were checked every 3–4 days for germinated seeds (i.e., radicle at least as long as the seed) which were removed from the Petri dishes. Seeds infested with fungi were subjected to a pressure test using tweezers, and in the case of softness, they were removed from the Petri dish and counted as non-germinated. After the test period, the cumulative proportion of germination was calculated per Petri dish, i.e., germinated seeds divided by the number of seeds sown (Ranal and Santana, 2006), given as % ± standard error in the text. The calculation based on the number of seeds sown was done for comparability with the garden experiment.

For the species *S. cernua* and *S. blepharophylla*, no laboratory tests could be carried out due to seed scarcity. The same holds for single populations of *S. aizoides* (population aiz_W) and *S. stellaris* (population ste_W; Supplementary Table 2).

### Common Garden Experiment: Emergence, Survival of Growing Season and Overwinter Survival

Seeds were sown in gardening pots (7 × 7 cm) which were filled with a fine-grained alpine soil mixture (5:2:2:2:1 leaf mold:ground earth:peat:silicate sand:lava) obtained from the Botanical Garden Innsbruck. Pots were placed in the Botanical Garden on the 15 November 2016 and 6 January 2018 following seed collections in the summers of 2016 and 2017, respectively. Seed collections from both years experienced a winter period in the pots, i.e., natural cold-wet stratification to break seed dormancy (Schwienbacher et al., 2011; Tudela-Isanta et al., 2018). During the growing season, the pots were regularly watered and randomly reshuffled. Recordings on emergence (i.e., cotyledon development) in the common garden began after snow-melt on 17 March 2017 and 11 April 2018, respectively. Emergence was then checked once every 2 weeks during the whole growing season (see Supplementary Table 3 for numbers of emerged seeds). As the cotyledons of *Saxifraga* spp. are tiny, we used magnifying glasses to ensure that all emerged seedlings were recorded. Recording further included removing seedlings from pots using a soft tweezer and putting them into separate multi-pots (Ø 2.5 cm; same soil mixture and saturated with water) so that the emerged seedlings were not double-counted. The emergence experiment ended on 22 September 2017 and 25 September 2018, respectively, lasting for 187 days in 2017, and 168 days in 2018, respectively. The proportion of cumulative emergence was calculated from the amount of seeds sown (given as % ± standard error in the text), as it was not possible to gather the number of non-emerged seeds in the soil-filled pots.

The emerged seedlings were arranged in multi-pots with a maximum of five seedlings per multi-pot, originating from matching populations, and regularly watered. Here, the survival of the seedlings (i.e., survival until the end of the growing season; Fenner and Thompson, 2005) was monitored (counting of seedlings every 3 weeks, i.e., 14 times) until the end of September of the same year as emergence events (Supplementary Figure 2). Survival in the growing season (“gs”) is expressed as the proportion of seedlings that survived from the number of seeds that emerged (% ± standard error in the text) on 19 October 2017 and 22 November 2018, respectively.

The seedlings that survived across the growing season in the multi-pots were repotted into bigger pots (7 × 7 cm) due to natural seedling growth (on 19 October 2017 and 22 November 2018, respectively). A maximum of 50 individuals per population was repotted due to space limitations. Five individuals per population were pulled together in one pot, which was buried underground, and watered regularly until the first snowfall at the experiment site. The seedling survival of a winter period (i.e., overwinter “ow”) was determined after snowmelt in the following year (6 April 2018 and 4 April 2019, respectively). Survival “ow” is expressed as the proportion of survived seedlings from repotted survivors (% ± standard error and numbers in the text).

Average mean soil temperatures of the growing seasons (2-cm depth) were 17.3°C (2017) and 19.6°C (2018); average minimum temperatures were 10.8°C (2017) and 13.0°C (2018); and average maximum temperatures were 27.4°C (2017) and 29.9°C (2018; Supplementary Figure 2).

### Data Analyses

#### Responses of Widespread and Narrow-Ranged *Saxifraga* spp.

Seeds from 23 species and 75 populations were available for testing germination (*N* = 584 Petri dishes). We used generalized- and linear-mixed models (package “lme4” Bates et al., 2019) to analyze germination percentage and mean time to germination (MTG), respectively, in response to the fixed-factors distribution (“wide,” “narrow”), temperature (“warm,” “cold”), and the interaction term; species and population nested within species were set as the random effects. The model for germination percentage was set assuming a binomial distribution (Ranal and Santana, 2006) with a logit link. The model was not overdispersed or zero-inflated as tested via the package “DHARMa” (Hartig, 2020). MTG was analyzed with a Gaussian link; model assumptions were met.

The time that seeds need to germinate gives insights into the dynamics of the germination process. The MTG per Petri dish was calculated in EXCEL via formulas in Ranal et al. (2009) as the following:


$$M\,T\,G,M\,T\,E=\sum_{i=1}^{k}n_{i}\,t_{i}/\sum_{i=1}^{k}n_{i}$$

where *t*_*i*_ is the time from the start of the experiment to the *i*th observation; *n*_*i*_: number of seeds germinated in the time *i* (not the accumulated number, but the number corresponding to the *i*th observation), and *k*: last time of germination. Due to mathematical reasons, MTG cannot be calculated when germination is zero for a whole Petri dish (Ranal and Santana, 2006), which was excluded from the analyses.

Seeds from all 25 species and 79 populations were available for testing emergence (*N* = 236 pots). Subsequently, we calculated the mean time to emergence (MTE) using the same approach proposed for the calculation of MTG, but based on the number of seedlings that emerged in the particular time interval *i* instead of germinated seeds (Ranal et al., 2009). Emergence percentage and MTE were analyzed following the same approach as proposed for germination percentage and MTG data analyses, without the temperature factor. Emergence can be zero if no seedling is able to emerge, which was the case in the eight pots.

To test if the survival of seedlings until the end of the growing season (“gs”) differed between distributions, we built a generalized linear mixed model (binomial distribution and logit link) with distribution and year (2017 and 201’) as a fixed factor; species and population nested within species were set as a random effects; and model assumptions were met. We observed strong seedling mortality in the summer of 2018, and we had to include the year of observation to account for this effect. Survival percentage of the winter period (“ow”) was analyzed using a generalized linear mixed model (binomial distribution and logit link) with distribution as the fixed effect, species and population nested within species as random effect; model assumptions were met.

To assess information on the ecology of the species, we used ecological indicator values of Landolt et al. (2010) (Eco-values in Table 1). Ecological indicator values describe ecological and biological characteristics of species by values that represent a species’ preferred climatic (T, K, L; Table 1) and soil conditions (F, W, R, N, H, D; Table 1), and species’ life form strategies (LF, KS; Table 1). The values are based on long-term compilations of expert knowledge and experiments (field or laboratory). We analyzed each response variable (germination percentage, MTG, emergence percentage, MTE, survival “gs” and survival “ow” percentages) as described above and included the 11 ecological indicator values (Table 1) individually in the models; likelihood ratio tests were used to assess a significant contribution of the values in the model. We then included those values that were significant in individual models to combined models. Ecological indicator values that were found to have a significant effect in the combined models were kept.

To determine the variance explained by fixed effects in the models, R^2^ for the null model (R^2^m0) and the conditional (full) model (R^2^m1), respectively, were calculated, according to Nakagawa and Schielzeth (2013). Furthermore, we extracted the variance components of the models (i.e., random factors) to determine the intraclass correlation coefficient [ICC; variance of random terms divided by variance of random term plus residual variance; Nakagawa et al. (2017)]. For more detailed information on model building and calculation on ICC (see Supplementary Text).

#### Intraspecific Variation

To assess whether or not the intraspecific variability of recruitment traits differs between widespread and narrow-ranged species, we used the coefficient of variation (CV), which is used as a measure for variation within or between groups (ratio of the standard deviation to the mean; Pearson, 1894; Lande, 1977; Meier and Holderegger, 1998). The CV is here expressed in its ratio form but can be interpreted as a percentage of variation (ratio times 100). Low values are considered as low variation whereas high values indicate high variation. The CVs of the response variables, namely germination percentage, MTG, emergence percentage, and MTE were calculated for each species. The basis for calculations were Petri dishes and pots, respectively, representing the variation within the Petri dishes or pots. Subsequently, the means of these population CVs reflect the variation within a species (i.e., intraspecific variation). If the initial response variable was zero, a CV cannot be calculated. The species-level CVs were then used as variables in simple linear regressions, with distribution as an explanatory factor variable, using separate datasets for warm and cold temperature conditions in the case of germination percentage and MTG. Species that have only single populations were excluded from these analyses. We used a square root transformation of the CV as it can be zero (Pearson, 1894; Lande, 1977). The CV for MTG and MTE were calculated according to Ranal et al. (2009). In our dataset, it was not possible to calculate the CV for germination percentage and MTG for enough number of species in the warm temperature to perform a meaningful analysis for this temperature setting.

To evaluate variations in temperature at the initial seed collection sites, we extracted mean annual temperatures from raster datasets available from the University of East Anglia Climatic Research Unit (Harris et al., 2021) for the years of seed collection 2016 and 2017, respectively (Supplementary Table 1). We used QGIS to extract values per location (via latitude and longitude). From these mean annual temperatures per population, we calculated CVs of temperature for individual species. These CVs were analyzed in a linear regression with distribution as the explanatory factor variable to assess if seed source temperature variation differs between distributional groups. Species with only single populations were excluded from these analyses.

#### Links Between Early Life Stages

To estimate the influence of the earliest transition phases on the subsequent one, we performed correlations on germination-emergence and emergence-survival “gs” (Spearman Method) on means per species of widespread and narrow-ranged species, i.e., mean germination (warm and cold) and the emergence of 23 species (data from 2017 and 2018); and emergence and the survival “gs” of summer 2017 of 17 species.

We used R software (R Core Team, 2020) and RStudio (RStudio Team, 2020) for analyses and graphs (“pastecs” Grosjean et al., 2018; “cowplot” Wilke, 2018; “GerminaR” Lozano-Isla et al., 2019; packages within “tidyverse” Wickham et al., 2019; “scales” Wickham and Seidel, 2020).

## Results

### Responses of Widespread and Narrow-Ranged *Saxifraga* spp.

**FIGURE 2:**
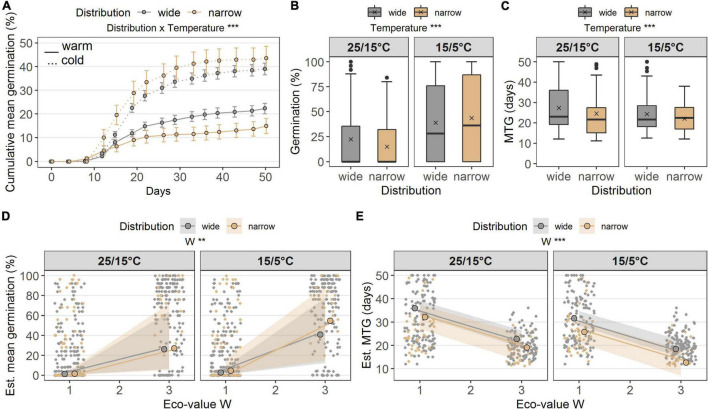
**(A)** Cumulative germination (%) in the laboratory at two temperature regimes (warm 25/15°C, cold15/5°C), grouped according to distribution ranges (wide, narrow). Points denote the cumulative mean on each day of observation, error bars denote ± standard errors. Boxes in **(B)** germination (%) and **(C)** MTG (days) illustrate the interquartile range with the median as line; whiskers illustrate the minimum and maximum; points denote outliers; x denotes the mean. Points without border in **(D)** germination (%) and **(E)** MTG denote the observed values colored according to the distribution range along the ecological indicator value W (alternate soil humidity); points with border denote the estimated means and colored ribbons denote confidence intervals from the models. Note different maxima on *y*-axis between panels. Stars denote significances at α = 0.01 (**) and α = 0.001 (***).

Seed germination was similar between widespread and narrow-ranged species under both temperature conditions (Figures 2A,B). Average germination percentage under the warm temperature was lower (wide: 22.3 ± 2.3%; narrow: 14.9 ± 3.2%) than under the cold temperature conditions (wide: 39.0 ± 2.5%; narrow: 43.6 ± 5.0%). Distribution *per se* did not affect germination percentage; however, the interaction of distribution × temperature did (*p* < 0.001, df = 1; Table 2A). Under cold temperature, total germination percentage was significantly greater than under warm temperature (*p* < 0.001, df = 1; Table 2A and Figures 2A,B). The MTG between the distribution groups was similar (Figures 2A,C). Under the warm temperature, average MTG was higher (wide: 27.3 days ± 1.1 standard error; narrow: 24.6 ± 2.8 days) than under the cold temperature (wide: 24.3 ± 0.7 days; narrow: 22.1 ± 1.0 days). Distribution was very weakly responsible for these results (*p* = 0.076), while temperature had a strong significant effect on MTG (*p* < 0.001, df = 1; Table 2B).

**TABLE 2:** Results of mixed models of studied recruitment traits (A) germination percentage, (B) mean time to germination (MTG), (C) emergence percentage, (D) mean time to emergence (MTE), (E) survival percentage after the growing season “gs,” and (F) overwinter survival percentage (“ow”), in response to distribution, temperature conditions, the interaction term, and ecological indicator values (see Table 1) that were found to be significant when individually added to the models.

Model terms	Full model (m1)	Chisq (m1)	*p*-value (m1)
**(A) Germination percentage**	Log-Odds: Est. (95% CI)		
Distribution	0.05 (−1.63, 1.72)	0.12	0.732
Temperature	0.68 (0.59, 0.77)	378.72	<0.001
Eco-value W	1.60 (0.69, 2.51)	9.42	<0.01
Distr × Temp°	0.51 (0.31, 0.71)	26.64	<0.001
**(B) MTG**	Est. (95% CI)		
Distribution	−3.77 (−9.39, 1.85)	3.15	0.076
Temperature	−4.30 (−5.46, −3.15)	46.02	<0.001
Eco-value W	−6.56 (−8.82, −4.30)	16.81	<0.001
Distr × Temp°	−2.05 (−5.00, 0.90)	1.85	0.173
**(C) Emergence percentage**	Log-Odds: Est. (95% CI)		
Distribution	−0.14 (−0.75, 0.47)	0.21	0.646
Eco-value H	−0.43 (−0.72, −0.15)	7.70	<0.01
Eco-value LF (h/t)	0.14 (−0.65, 0.92)/−2.71 (−3.78, −1.65)	17.96	<0.001
**(D) MTE**	Est. (95% CI)		
Distribution	−0.32 (−0.92, 0.28)	1.10	0.295
Eco-value T	0.61 (0.26, 0.96)	9.35	<0.01
Eco-value H	0.49 (0.22, 0.75)	9.32	<0.01
**(E) Survival percentage “gs”**	Log-Odds: Est. (95% CI)		
Distribution	0.62 (−1.23, 1.48)	1.85	0.174
Year garden (2017)	−2.70 (−3.16, −2.24)	80.08	<0.001
**(F) Survival percentage “ow”**	Log-Odds: Est. (95% CI)		
Distribution	−0.25 (−0.50, 0.00)	3.48	0.062
Eco-value F	−0.38 (−0.51, −0.25)	11.25	<0.001

*All models were set with distribution “wide” as reference, temperature “warm” in cases (A,B), LF “c” in case of (C). Est., Estimate; CI, Confidence intervals; Chisq, test statistic and p-values of full model (m1). Chi-square and p-values were estimated with maximum likelihood; ^°^in cases of interactions we used contrast-coding (Levy, 2018).*

**FIGURE 3:**
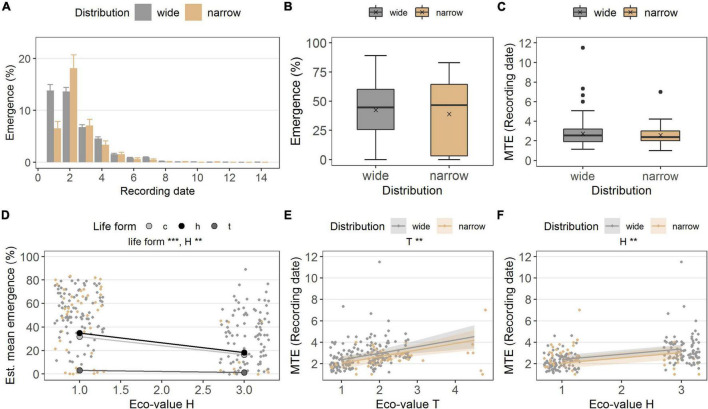
**(A)** Emergence percentage in the common garden, grouped in distribution (wide, narrow), at each recording date (i.e., every 2 weeks). Bars denote mean at each recording date, error bars denote ± standard errors. Boxes in **(B)** emergence percentage and **(C)** MTE (recording date) illustrate the interquartile range with the median as line; whiskers illustrate the minimum and maximum; points denote outliers; x denotes the mean. Points without border in **(D)** emergence percentage **(E,F)**, MTE, denote the observed values colored according to distribution along ecological indicator values H (humus) and T (Temperature value); points with border denote the estimated means per ecological indicator value life form (c, chamaeophyte; h, hemicryptophyte; t, therophyte), and colored ribbons denote confidence intervals from the models [missing in **(D)** to simplify the graph]. Note different maxima on *y*-axis between panels. Stars denote significances at α = 0.01 (**) and α = 0.001 (***).

In the common garden, the two distribution groups emerged similarly (*p* = 0.646, df = 1; Table 2C and Figures 3A,B). Widespread species’ emergence was 42.3 ± 1.7% on average, while narrow-ranged species’ emergence was 39.0 ± 3.9% (Figures 3A,B). The MTE also did not differ between the distribution groups (*p* = 0.295; Table 2D and Figure 3C). Widespread species emerged on average after 38.1 ± 1.3 days (MTE) and narrow-ranged species after 36.1 ± 2.0 days (MTE).

**FIGURE 4:**
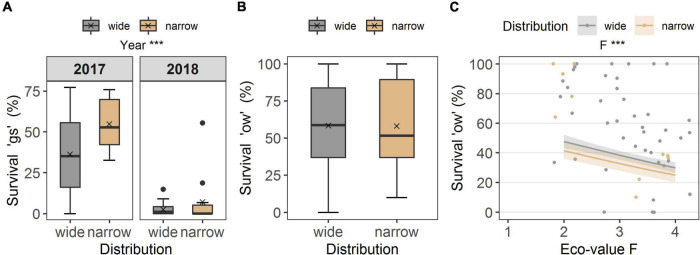
**(A)** Seedling survival of the growing season (“gs”) in the common garden, grouped according to distribution (wide, narrow), for the years of observation (2017, 2018), and **(B)** overwinter seedling survival (“ow”). Boxes in **(A)** survival “gs” and **(B)** survival “ow” illustrate the interquartile range with the median as line; whiskers illustrate the minimum and maximum; points denote outliers; x denotes the mean. Points without boarder in **(C)** survival “ow” denote the observed values colored according to distribution along the ecological indicator value F (humidity), and colored ribbons denote confidence intervals from the model. Note different maxima on *y*-axis between panels. Stars denote significances at α = 0.001 (***).

**FIGURE 5:**
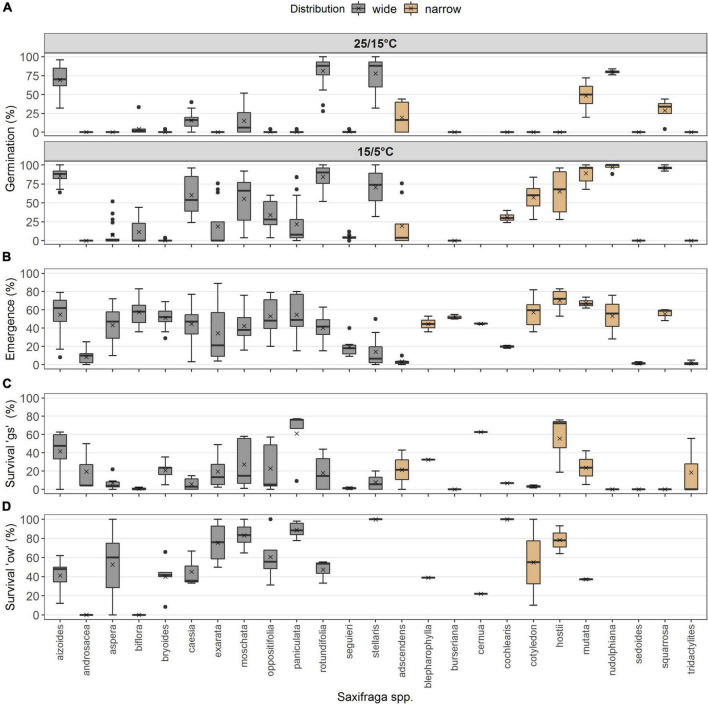
Recruitment responses at species level. **(A)** Germination percentage under warm and cold laboratory temperature (*N* = 584). **(B)** Emergence percentage in the common garden under natural seasonal climate (*N* = 236). **(C)** Survival percentage “gs” (*N* = 79), and **(D)** survival percentage “ow” (*N* = 52). Species are listed in alphabetical order within each distribution. Boxes illustrate the interquartile range with the median as line; whiskers illustrate the minimum and maximum; points denote outliers; x denotes the mean.

No differences occurred between the two distribution types in survival “gs,” i.e., to the end of the growing season (*p* = 0.174, df = 1), but the year of observation made a strong difference (*p* < 0.001, df = 1; Table 2E). Seedling survival “gs” in the common garden was on average 36.2 ± 4.2% in 2017 for widespread and 54.7 ± 7.3% for narrow-ranged species (Figure 4A). In summer 2018, only 2.6 ± 0.7% of seedlings from wide and 7.11 ± 4.3% of seedlings from narrow-ranged species survived the growing season (Figure 4A). The survival of the winter period “ow” was also similar for both the distribution groups (*p* = 0.062, df = 1), i.e., 58.4 ± 4.6% for widespread and 54.6 ± 10.1% for narrow-ranged species (Figure 4B). Recruitment responses at the species level are presented in Figure 5.

The ecological indicator value W (alternating soil humidity) significantly affected the germination percentage (*p* < 0.01, df = 1, Table 2A), with species assigned to higher *W*-values showing a higher germination percentage (Figure 2E). Alternating soil humidity (W) also significantly impacted MTG (*p* < 0.001, df = 1, Table 2B), which decreased for species having higher *W*-values (Figure 2E). Moreover, species with lower emergence tended to have high indicator value H (humus content; *p* < 0.01, df = 1; Table 2C and Figure 3D). Furthermore, the emergence differed among life forms (Figure 3D), with therophytes (two species) significantly emerging less than herbaceous chamaephytes and hemicryptophytes (Table 2C and Supplementary Table 4). The MTE was significantly enlarged in response to the ecological indicator values T (temperature; *p* < 0.01, df = 1) and H (humus content; *p* < 0.01, df = 1, Table 2D and Figures 3E,F). None of the ecological indicator values were significant in the model for survival “gs” (Table 2E), but the ecological indicator value F (soil moisture) played a significant role for survival “ow” (*p* < 0.001, df = 1; Table 2F and Figure 4C), i.e., species with high *F*-value had low survival “ow.”

Variance components of the models are listed in Supplementary Table 5 and are illustrated in Supplementary Figure 3. The full model of germination percentage accounted for 67.30% (R^2^m1) of the variance and 22.63% was due to the fixed factors (R^2^m0; Supplementary Table 5A). The random factor level species had a variance of 40.3% and a population of 17.5% (Supplementary Figure 3 and Supplementary Table 4A). The full model of MTG accounted for 84.4% of the variance and 42.1% was due to fixed effects. Random effects, i.e., species (6.7%) and populations (66.4%), also accounted for a substantial fraction of variance (Supplementary Figure 3 and Supplementary Table 5B). The full model of emergence percentage accounted for 26.4% of the variance, but only 0.5% was due to fixed-factor distribution (Supplementary Figure 3 and Supplementary Table 5C). The random factor level species had 6.1% of the variance in the model and a population of 8.0% (Supplementary Figure 3 and Supplementary Table 5C). The full model survival “gs” accounted for 46.9% of the variance, and 28.7% was due to fixed factors (Supplementary Table 5E). The random factor level species made up for 16.7% of variance, and population 8.9% (Supplementary Figure 3 and Supplementary Table 5E). The full model of survival “ow” accounted for 2.7% of the variance, and 2.4% was due to the fixed factors (Supplementary Table 5F). The random factor level species had 0.0% and a population of 0.3% of the variance in the model, while the largest portion of the variance in survival “ow” remained unexplained (Supplementary Figure 3 and Supplementary Table 5F).

### Intraspecific Variation

**FIGURE 6:**
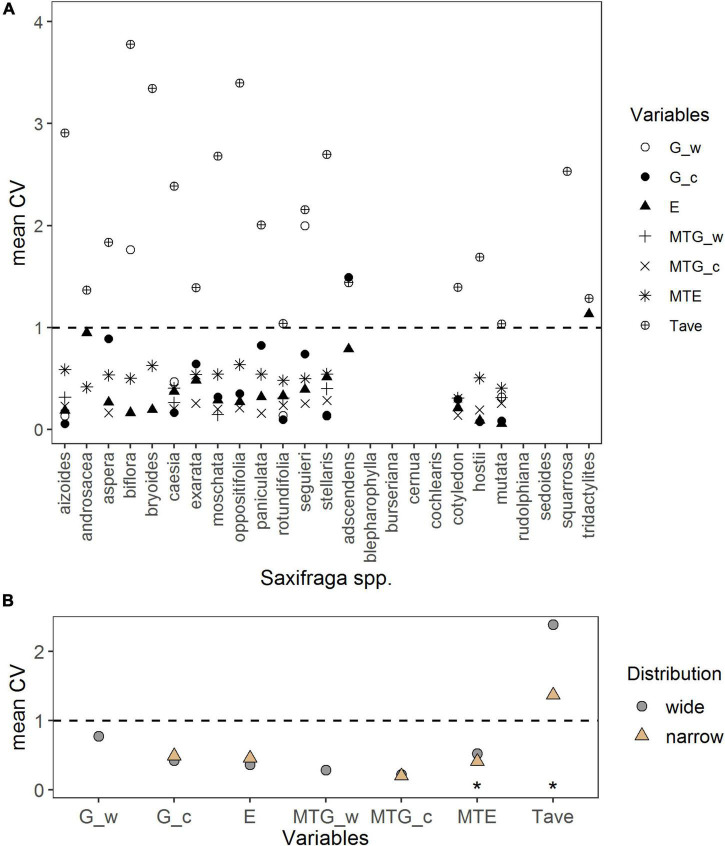
**(A)** Intraspecific variation per species represented by the coefficient of variation (CV) of response variables: Germination percentage in warm (G_w) and cold (G_c) temperature in the laboratory, emergence percentage (E), mean time to germination in warm (MTG_w) and cold (MTG_c) temperature, mean time to emergence (MTE), and average annual temperature at the seed collection sites (Tave). **(B)** Comparison of mean CV of response variables per distribution range, done by linear regressions. Note different maxima on *y*-axis between panels. Stars denote significances at α = 0.05 (*).

Intraspecific variation of response variables per species is illustrated in Figure 6A. Intraspecific variation of germination percentage under warm temperature ranged from lowest CV = 0.13 (*S. aizoides* and *S. stellaris*) to highest CV = 2 (*S. seguieri*); the only one value for narrow-ranged species (CV = 0.31 *S. mutata*) prevented further analyses. Under the cold temperature, intraspecific variation of germination percentage ranged from lowest CV = 0.06 (*S. aizoides*) to highest CV = 1.49 (*S. adscendens*). For the MTG under warm temperature, CV ranged from 0.14 (*S. moschata*) to 0.40 (*S. stellaris*) and under the cold temperature from 0.14 (*S. cotyledon*) to 0.29 (*S. stellaris*). Intraspecific variation of emergence was lowest in *S. mutata* (CV = 0.06) and highest in *S. tridactylites* (CV = 1.13). Intraspecific variation of MTE was lowest for *S. cotyledon* (CV = 0.31) and highest for *S. oppositifolia* (CV = 0.64). When analyzing CVs to assess intraspecific variation between distribution groups (Figure 6B), narrow-ranged species had a significantly lower intraspecific variation in MTE (*p* = 0.02; Supplementary Table 8) than widespread species. There were no significant intraspecific variations in germination percentage, MTG, and emergence percentage between the two distributional groups (*p* > 0.4; Figure 6B and Supplementary Table 6).

Intraspecific variation of the average annual temperatures at seed collection sites ranged from lowest CV = 1.04 (*S. mutata*) to highest CV = 3.79 (*S. biflora*). It differed significantly between distribution groups, with narrow-ranged species having significantly lower temperature variation at their sites of seed collection (*p* = 0.017; Supplementary Table 6) than the widespread species.

### Links Between Early Life Stages

Seed germination and seedling emergence followed a trend of a positive correlation, but only germination percentage in cold conditions and emergence of narrow *Saxifraga* spp. was significant (*R* = 0.718, *p* = 0.019). Conversely, emergence and survival “gs” were significantly positively correlated in widespread species (*R* = 0.685, *p* = 0.014; Supplementary Table 7).

## Discussion

In this study, we investigated early life stages of 25 *Saxifraga* spp. from the European Alps, representing species with wide and narrow geographic ranges. By conducting laboratory and common garden experiments, we studied recruitment transition phases, i.e., germination, emergence, and survival (first growing season and overwinter).

Germination was triggered by the cool temperature conditions in the laboratory in species from both geographic ranges, leading to higher germination percentages with faster MTG in this setting. However, because of the limited options for studying temperature requirements for germination (i.e., only two regimes), further research is needed to better define the germination temperature niche. Yet, these results are in line with Giménez-Benavides and Milla (2013) who reported that cold temperatures favored the germination of two altitudinal vicariant *Saxifraga* spp., independent of their altitudinal occurrence. Similarly, Di Cecco et al. (2019) recorded increased germination under cool temperatures compared with warmer ones in *S. italica*. These results contrast with the general assumption that germination of alpine species is enhanced by warm temperature cues (Walder and Erschbamer, 2015; Fernández-Pascual et al., 2020; Vázquez-Ramírez and Venn, 2021). As germination is a key process for population dynamics (Donohue et al., 2010; Larson et al., 2015; Rosbakh and Poschlod, 2015; Rosbakh et al., 2020), enabling populations to migrate and colonize new habitats, it is crucial for population persistence (Cochrane et al., 2015). Current and future scenarios of warming (IPCC et al., 2021) pose a threat to Alpine *Saxifraga* spp., i.e., warming may lead to reduced germination, which in the long-term may cause a decline in *Saxifraga* populations.

In our laboratory experiments, there was a significant distribution x temperature interaction, with widespread species germinating more at warm temperatures than narrow-ranged species. In combination with their germination in the cold setting, we may interpret these results as widespread species having an overall broader germination temperature niche. A wider germination niche may expose seedlings to the unfavorable post-germination conditions (Donohue et al., 2010; Mondoni et al., 2015; Rosbakh et al., 2020), but may also increase opportunities for recruitment in a diversity of habitats. Consequently, a wider regeneration niche (beginning with germination) could have contributed to a wider distribution range of widespread *Saxifraga* spp., thereby supporting our first hypothesis. In the literature, contradicting results regarding niche breadth and distribution ranges are found (Finch et al., 2018). For example, Thompson et al. (1999) tested the germination niche of 263 herbaceous plants in central England (United Kingdom) and found no correlation of germination niche breadth with range size, although the range size correlated with the temperatures under which species could germinate. In contrast, more recently, Rosbakh et al. (2020) could define five distinct germination niches along a hydroperiod gradient, showing that seeds were able to select a niche that favors seedling survival.

Cold temperature triggered a faster germination in the studied *Saxifraga* spp., and the ecological indicator value for alternating soil humidity (W) made a significant contribution in the models for germination percentage and MTG. Species with high values of W germinated faster and to higher extents, regardless of their distribution range. This can be an important survival strategy in alpine species, especially for those inhabiting bare ground soils that have a low water-holding capacity, but where many *Saxifraga* spp. normally thrive. Under these conditions, species with a fast germination may benefit from the available soil moisture after snowmelt (Giménez-Benavides et al., 2007; Mondoni et al., 2015), meaning a reduced risk of seedlings emergence under possible drought conditions in summer (Garnier et al., 2021).

Widespread and narrow-ranged species had similar emergence percentages and MTE in the common garden experiment. On the other hand, emergence phenology differed in species depending on their ecological indicators for humus content (H), with a higher and faster emergence for those with low H-values. This suggests that *Saxifraga* spp. assigned to medium humus content (i.e., *H* = 3; *n* = 10) could have had difficulties to recruit under the fine-grained alpine soil mixture. However, these latter species have their main occurrences in upper subalpine and alpine vegetation belts (see ecological indicator values for temperature in Table 1), where fine-grained soils occur naturally. Another result was that emergence differed among life forms. Surprisingly, the two therophytes in the dataset emerged at significantly lower percentages than herbaceous chamaephytes and hemicryptophytes, although therophytes usually emerge well due to their short life cycle (Fenner and Thompson, 2005; Landolt et al., 2010). As a possible explanation, we suggest that light requirements for germination and subsequent emergence were not met in the common garden. Indeed, although seeds in the garden were exposed to daily sunlight fluctuations, the possibility that they were buried and, therefore, experienced darkness, cannot be ruled out. Photoinhibition of seed germination was noted as a prominent feature in therophytes and small-seeded plants as a strategy to build a permanent soil seed bank (Saatkamp et al., 2011; see Carta et al., 2017 and references therein). Furthermore, some therophytes need disturbances for seed germination and emergence, such as an indirect effect altering the light conditions (Saatkamp et al., 2011).

The MTE for narrow-ranged and widespread species did not differ (i.e., 36–38 days, respectively), thereby showing a similar emergence phenology across the species tested, and reflecting similar emergence times as previously found in other *Saxifraga* spp. in the wild (Mondoni et al., 2015). Emergence times of +30 days do not reflect fast germination after snowmelt, rather it indicates seed dormancy. This slow germination may reflect a bet-hedging strategy for spreading the germination overtime, and with it the risk of seedling mortality (Ooi et al., 2009; Saatkamp et al., 2011). However, in the model of MTE, the ecological indicator value for temperature (T) was significant, with truly alpine species (low T-values) emerging faster than species assigned to higher T-values. These results support previous findings that alpine species germinate and emerge fast after snowmelt to make the best use of soil moisture and avoid the onset of summer drought (Giménez-Benavides et al., 2007; Mondoni et al., 2015; Garnier et al., 2021). Another aspect is that the fast emergence of alpine species could be a strategy to cope with the shorter growing seasons compared with that at lower elevations (Mondoni et al., 2020).

Seedling survival of the first growing season did not differ between narrow-ranged and widespread species, and also no ecological indicator values were found to be significant in the model. Hence, our findings for this transition stage do not support our hypothesis. Seedling survival was significantly low during the second experiment onset (i.e., summer 2018). We attribute the high seedling mortality of this year to drought stress induced by methodological aspects, as seedlings are small and particularly vulnerable to drought stress in the first growing season (Mondoni et al., 2020; Garnier et al., 2021). The multi-pots used for survival monitoring were regularly watered, but also dried out fast in the exceptionally hot summer of 2018 in Europe (soil temperature measurements showed +2.3°C annual mean temperature in the Botanical Garden compared with summer 2017). Although we did not monitor soil water potential, we believe that the species faced drought stress in the garden experiment in the second onset in 2018. Drought stress has been identified to severely reduce the emergence and increase seedling mortality (Garnier et al., 2021; Vázquez-Ramírez and Venn, 2021), especially in unprotected open microhabitats without shelter from surrounding vegetation (Stöcklin and Bäumler, 1996; Margreiter et al., 2021), as found on bare ground or in rock crevasses.

Subsequently, survival into the second growing season (“ow”) was similar between distribution ranges, and the ecological indicator value for soil moisture was significant, with species assigned to higher levels of soil moisture surviving significantly less. These results certainly reflect unsuitable water conditions for the species in the garden, but this part of the study should be viewed with caution due to the repeated transplanting of seedlings.

All transition stages varied species-specifically, and it was not possible to draw a solid pattern regarding the distributional range of the species. Accordingly, many studies recorded interspecific variation of seedling emergence in the field (Münzbergová, 2004; Meineri et al., 2013; Mondoni et al., 2015, 2020; Margreiter et al., 2021), and differences in plant performances occurred even if species were phylogenetically closely related (e.g., Lavergne et al., 2004; Milla et al., 2009; Rosbakh et al., 2020).

We found substantial intraspecific variation in germination, emergence, and their mean times to germinate/emerge (Figure 6A). At the intraspecific level, narrow-ranged species had a significantly lower variation of MTE than widespread species. Such time constrain for seedling emergence of narrow-ranged species could reflect narrower suitable climatic conditions for this stage. In this regard, mean annual temperature variations between collecting sites were significantly lower in narrow-ranged compared with widespread species, indicating a link between the timing of emergence and climate variability at the species’ home sites. Intraspecific variation in seed traits may be linked to a set of climatic conditions that possibly reflect local adaptations (Cochrane et al., 2015). For example, Giménez-Benavides et al. (2007) reported favoring effects of local adaptation for seedling emergence in remnant populations of *Silene ciliata*, and Cotado et al. (2020) found that intraspecific variation in dormancy levels of *Saxifraga longifolia* (Pyrenees) was linked to climatic conditions at the seed collection sites. Furthermore, intraspecific variation in seed traits was found along germination temperature gradients from different locations (Walter et al., 2020). Clearly, the timing of emergence (and germination) is driven by temperature (Walck et al., 2011; Cochrane et al., 2015; Anderson and Wadgymar, 2020). A diverse emergence timing can lead to the spread of emergence events over a broader time scale, spreading the risk of hazards in post-germination phases (e.g., frost, drought, herbivores; Larson and Funk, 2016). Variation in germination timing was recognized as an important variable for natural selection in *Arabidopsis* (Donohue et al., 2005). Genotypes that had a high variation of germination timing showed a higher fitness due to the postponement of germination toward an optimum for growth, while genotypes with low variation were lacking such opportunities for selection (Donohue et al., 2005). Carrying forward these ideas of germination timing to emergence timing, the greater variation in emergence timing of widespread species found here could have favored their wider distribution range in the past and may be advantageous while facing climate changes when rapid adaptations are needed to cope with novel climatic conditions. Our hypothesis on a greater intraspecific variation of germination and emergence responses for widespread species can, however, only be partially confirmed (i.e., emergence timing).

Research to date suggests that recruitment success may be driven by different seed-trait relationships, showing that, for example, seed mass positively correlates with seed germination (Pearson et al., 2002; Kahmen and Poschlod, 2008) or that dispersal traits positively correlate with seedling emergence (Chen and Giladi, 2020). Relationships between transition stages within recruitment are less studied but suggest that recruitment success may depend on early demographic processes, such as germination and emergence (Fraaije et al., 2015; Larson et al., 2015). In our study, germination and emergence differently affected subsequent transitions phases depending on species distribution range, with emergence probabilities being a strong predictor of survival only for widespread species. Further research is therefore needed to identify seed and seedling functional traits driving such variations between distribution groups.

Finally, we address a methodological point that may have influenced the outcome of this study. The number of populations between widespread and narrow-ranged species was unbalanced, but to the best of our knowledge the mixed models used are well equipped to deal with unbalanced or missing data in the data matrix (Nakagawa and Schielzeth, 2013; Bates et al., 2019). Nevertheless, narrow-ranged species are by definition less common, and we assumed that narrow-ranged species have a narrower niche, reflected by lower recruitment responses and lower intraspecific variability. Our hypotheses were partly supported by the results; hence the outcome of analyses may change to a clearer picture if we had a greater number of populations of narrow species.

## Conclusion

In summary, recruitment traits of widespread and narrow-ranged species were similar in five out of six cases (i.e., MTG, emergence percentage, MTE, survival “gs” and “ow”). Germination percentage in warm laboratory temperature was higher for widespread than for narrow-ranged species (interaction of distribution × temperature), indicating a higher tolerance of warm temperatures for the former, which may have facilitated their occupation of a greater variety of habitats or habitats across a larger geographical range, respectively. However, these results should be viewed with caution as we only tested germination in two temperature regimes. Intraspecific variation of MTE differed between widespread and narrow-ranged species, and so did the mean annual temperatures between the respective seed collection sites. These results indicate a relationship between climatic cues and recruitment responses. Ecological indicator values did not contribute to explaining the recruitment differences between species distribution ranges, but indicators of soil components underlined that they act as driving factors, which need further investigation in *Saxifraga* spp. In conclusion, our findings on recruitment traits of widespread and narrow-ranged *Saxifraga* spp. suggest that the differences between the distributional groups are mostly due to germination differences, while subsequent life-transition stages play a minor role. The overall recruitment niche of *Saxifraga* spp. shown here, characterized by low-temperature requirements and sensitivity to drought stress, highlight severe challenges in a warmer climate, especially for narrow-ranged species.

## Data Availability Statement

The data used in the study are included in the article/Supplementary Material, further inquiries can be directed to the corresponding author/s.

## Author Contributions

VM and BE designed the study. VM collected experimental data. VM performed data analyses with contributions of FP. VM led manuscript writing with substantial contributions from BE and AM. All authors performed seed collections and contributed to the interpretation of results.

## Conflict of Interest

The authors declare that the research was conducted in the absence of any commercial or financial relationships that could be construed as a potential conflict of interest.

## Publisher’s Note

All claims expressed in this article are solely those of the authors and do not necessarily represent those of their affiliated organizations, or those of the publisher, the editors and the reviewers. Any product that may be evaluated in this article, or claim that may be made by its manufacturer, is not guaranteed or endorsed by the publisher.

## Footnotes

1
http://geocat.kew.org/editor

2
http://www.alpineseedconservation.eu/
