# Supplementary material for: Recruitment Traits Could Influence Species’ Geographical Range: A Case Study in the Genus Saxifraga L
Source: Front Plant Sci. 2022 May 13;13:827330. doi: 10.3389/fpls.2022.827330 (PMC9136331; doi:10.3389/fpls.2022.827330)
Supplement: Supplementary file 1 [file Data_Sheet_1.pdf]

# *Supplementary Material*

## 1 Supplementary Tables

**Supplementary Table S1:** Study species of the genus *Saxifraga* (Saxifragaceae), populations collected in the Alps (Southwest SW, West W, Central C, East E), Latitude and Longitude (WGS 84 coordinate system), Altitude in meters above sea level (a.s.l.), date of seed collection, annual average temperature (Tave) and standard error (sd) at the seed collection sites in the matching year, and description of collection sites.

| *Saxifraga* spp. | Populations | Latitude (N) | Longitude (E) | Altitude (a.s.l.) | Date collection | Yearly Tave (°C ± Sd) | Collection site |
|---|---|---|---|---|---|---|---|
| *adscendens* | ads_C | 46.833389 | 11.046444 | 2617 m | 02/09/2016 | 3.84 ± 6.05 | Obergurgl, Hohe Mut, Hangseite Rotmoostal, Ötztal, Tirol |
| | ads_SW | 44.614171 | 6.811867 | 2458 m | 27/08/2017 | 4.95 ± 6.47 | Lac Sainte-Anni. Ceillac 05600 |
| *aizoides* | aiz_SW | 44.719005 | 6.903441 | 2023 m | 07/08/2017 | 4.95 ± 6.47 | Col Agnel, Hautes-Alpes |
| | aiz_W | 46.201759 | 7.510229 | 2183 m | 14/09/2016 | 1.33 ± 5.74 | L´Ar du Tsan, Mont-Noble, Wallis |
| | aiz1_C | 46.826722 | 11.046222 | 2479 m | 09/09/2016 | 3.84 ± 6.05 | Weg Stempelstelle Rotmoostal Richtung Rotmoos, Obergurgl, Ötztal, Tirol |
| | aiz1_E | 47.561111 | 14.6525 | 1700 m | 13/09/2016 | 7.18 ± 7.08 | Heßhütte, Richtung Zinödl, Nationalpark Gesäuse, Steiermark |
| | aiz2_C | 46.520611 | 10.409167 | 2200 m | 28/08/2016 | -0.65 ± 5.40 | 2° Casa Cantoniera per Passo Stelvio |
| | aiz2_E | 47.60055 | 14.05214 | 1560 m | 06/09/2016 | 6.95 ± 6.70 | Südseite Schwarzensee, Liezen, Bad Mitterndorf Tauplitzalm, Steiermark |
| *androsacea* | and_C | 46.821483 | 11.051433 | 2698 m | 12/09/2016 | 3.84 ± 6.05 | Weg Hohe Mut Richtung Stempelstelle, Rotmoostal, Obergurgl, Ötztal |
| | and_E | 47.38077 | 14.16783 | 2150 m | 20/07/2016 | 5.21 ± 6.38 | Donnersbach, Planneralm, Schreinl-Gipfel, Wölzer Tauern, Steiermark |
| | and_SW | 44.685076 | 6.974816 | 2636 m | 07/08/2017 | 4.95 ± 6.47 | Molines en Queyras, Hautes-Alpes |
| *aspera* | asp_C | 46.86625 | 11.007833 | 2148 m | 19/08/2016 | 3.84 ± 6.05 | Hangseite Beilstein, Am Beil, oberhalb Brücke, Obergurl, Ötztal, Tirol |
| | asp1_SW | 45.462722 | 7.137667 | 2200 m | 07/08/2017 | 6.47 ± 6.69 | Valle Orco, comune di Ceresole Reale, chiesetta nei pressi del Lago Serrù |
| | asp1_W | 46.249028 | 7.841812 | 1960 m | 07/10/2016 | 1.33 ± 5.74 | Hannig, Törbel, Wallis |

| | | | | | | | |
|---|---|---|---|---|---|---|---|
| | asp2_SW | 44.752418 | 6.258782 | 1415 m | 02/07/2017 | 5.87 ± 6.53 | Champoléon, Hautes-Alpes |
| | asp2_W | 46.209425 | 8.0624837 | 2158 m | 17/08/2017 | 3.98 ± 6.55 | Glatthorn, Simplon, Rouches en bordure de la route au pied Sud du Glatthorn |
| | asp3_W | 45.979275 | 7.782536 | 2740 m | 28/07/2017 | 4.96 ± 6.62 | Gornergrat, Zermatt, Usser Gornerli |
| *biflora* | bif_C | 46.973182 | 12.280486 | 2601 m | 05/10/2017 | 0.77 ± 6.57 | Großbachtal, Osttirol, Grube Nähe Neuer Reichenbergerhütte |
| | bif_SW | 45.062912 | 6.41417 | 2697 m | 31/07/2017 | 5.98 ± 6.63 | Crêtes du Galibier, Savoie, Face du Sud de la Pierre Avoi |
| | bif_W | 46.209416 | 8.062484 | 2730 m | 23/08/2017 | 3.98 ± 6.55 | Hirli, Zermatt, Face du nord du haute de la moraine sous Hirli |
| *blepharophylla* | ble_E | 47.38077 | 14.16783 | 2150 m | 20/07/2016 | 5.21 ± 6.38 | Schreinl Gipfel, Donnersbach, Planneralm, Wölzer Tauern, Steiermark |
| *bryoides* | bry_E | 47.28333 | 14.05461 | 2310 m | 30/08/2016 | 5.21 ± 6.38 | Sölkpass, Deneck, 550 m SE Deneck Gipfel |
| | bry_SW | 44.68569 | 6.979296 | 2703 m | 22/08/2017 | 4.95 ± 6.47 | Molines en Queyras, Hautes-Alpes |
| | bry_W | 46.129288 | 7.965835 | 2480 m | 20/09/2016 | 1.33 ± 5.74 | Mälliga, Saas-Grund, Wallis |
| | bry1_C | 46.839972 | 11.029583 | 2280 m | 21/09/2016 | 3.84 ± 6.05 | Rotmoostal Eingang, Obergurgl, Ötztal, Tirol |
| | bry2_C | 46.6935 | 10.168361 | 2300 m | 03/08/2016 | -0.65 ± 5.40 | Val Dosdè, Valdidentro (Sondrio), Lombardia, Italy |
| *burseriana* | bur_E | 47.5804 | 15.22576 | 1788 m | 28/06/2017 | 6.8 ± 8.04 | Aflenzer Bürgeralm, Schönleitenhaus, 21 km north of Bruck/Mur |
| *caesia* | cae_SW | 45.258 | 6.88345 | 2449 m | 23/08/2017 | 4.09 ± 6.47 | Mont Cenis - Savoie, Conbe de Cléry |
| | cae_W | 46.062327 | 9.05987 | 1564 m | 16/08/2017 | 4.73 ± 6.60 | Cima dell´Oress, Lugano, Eboulis des pentes N de la Coma del´Oress |
| | cae1_C | 47.311867 | 11.386817 | 2239 m | 11/09/2016 | 4.4 ± 6.12 | Nordkette, Goetheweg und Pfeishütte/Kreuzjöchl, Tirol |
| | cae1_E | 48.189065 | 16.382294 | 196 m | 31/08/2016 | 11.34 ± 7.70 | index no BK 20160831-7 |
| | cae2_C | 46.52061 | 10.409166 | 2200 m | 28/08/2016 | -0.65 ± 5.40 | 2° Casa Cantoniera per Passo Stelvio |
| | cae2_E | 47.6014 | 14.02459 | 1630 m | 07/09/2016 | 6.95 ± 6.70 | Tauplitzalm, calcareous talus ares "Steirerseeleiten", west slope of Sturzhahn |
| *cernua* | cer_W | 46.34082 | 7.477772 | 2700 m | 17/08/2016 | 2.65 ± 5.93 | Not available |
| *cochlearis* | coc_SW | 43.940482 | 7.519176 | 610 m | 17/08/2017 | 15.03 ± 6.30 | Breil sur Roya, Alpes maritimes |
| *cotyledon* | cot1_W | 46.129648 | 8.0961311 | 1762 m | 17/08/2017 | 3.98 ± 6.55 | Zwischbergen, Fah, Rocher en bordure de la route en aval du barrage de Fah |
| | cot2_W | 46.434667 | 9.340028 | 1390 m | 22/08/2017 | 4.73 ± 6.60 | Sondrio, comune di Madesimo, SP1 |
| *exarata* | exa_C | 46.835278 | 11.018056 | 3020 m | 03/09/2016 | 3.84 ± 6.05 | Hangerer Gipfel, Obergurgl, Ötztal, Tirol |
| | exa_W | 46.117953 | 7.2004417 | 2444 m | 13/08/2017 | 2.79 ± 6.42 | Bagnes, Pierre Avoi, Valais |

| | | | | | | | |
|---|---|---|---|---|---|---|---|
| | exa1_SW | 43.989667 | 7.65725 | 1900 m | 03/08/2016 | 15.04 ± 5.67 | Passo dell'Incisa, Saorge, Alpes-Maritimes, France |
| | exa2_SW | 44.68568 | 6.979295 | 2703 m | 22/08/2017 | 4.95 ± 6.47 | Molines en Queyras, Hautes-Alpes |
| *hostii* | hos_E | 47.35488 | 15.43922 | 1170 m | 29/07/2016 | 7.55 ± 7.32 | Fladnitz an der Teichalm, Weiz, Steiermark |
| | hos1_C | 46.62143 | 12.7201 | 1900 m | 17/08/2017 | 3.39 ± 6.89 | Belluno, comune di Sappada, footpath to Calvi hut |
| | hos2_C | 46.089253 | 11.842441 | 1969 m | 11/08/2016 | 2.9 ± 6.01 | Parco Nazionale Dolomiti Bellunesi, sovrafontane sovramonte, strada per rifugio dal Piaz (Belluno), Italy |
| *moschata* | mos_SW | 44.760606 | 6.285373 | 1503 m | 02/07/2017 | 5.87 ± 6.53 | Champoléon, Hautes-Alpes |
| | mos1_C | 47.312931 | 11.386515 | 2334 m | 15/09/2016 | 4.4 ± 6.12 | Hafelekarkreuz, Nordkette, Innsbruck |
| | mos1_E | 47.49841 | 14.92883 | 2114 m | 24/08/2017 | 5.6 ± 7.64 | Leoben, Eisenerzer Reichenstein, 350m west of Eisenerzer Reichenstein Hütte |
| | mos2_C | 46.973181 | 12.280485 | 2601 m | 05/10/2017 | 0.77 ± 6.57 | Großbachtal, Osttirol, Grube Nähe Neuer Reichenbergerhütte |
| | mos2_E | 47.62265 | 14.22689 | 1930 m | 19/08/2016 | 6.95 ± 6.70 | Angererkogel, Nordseite, Liezen, Steiermark |
| *mutata* | mut_C | 47.315083 | 11.213639 | 1435 m | 24/08/2016 | 4.4 ± 6.12 | Seefeld Reith, Bachschlucht SÖ Maximilianshütte |
| | mut_W | 46.0311585 | 6.48235507 | 1300 m | 21/08/2017 | 10.59 ± 7.18 | Mont-Saxonnex, Haute-Savoie |
| *oppositifolia* | opp_E | 47.27509 | 14.3549 | 2114 m | 16/07/2017 | 5.08 ± 7.23 | Murtal, Styria, Pusterwald Kleiner Zinken |
| | opp_SW | 44.631722 | 6.043333 | 2096 m | 12/07/2017 | 5.87 ± 6.53 | Pic de Gleize, Hautes-Alpes |
| | opp_W | 46.132008 | 7.967039 | 2467 m | 20/09/2016 | 1.33 ± 5.74 | Mälliga, Saas-Grund, Wallis |
| | opp1_C | 46.837472 | 11.045 | 2581 m | 09/09/2016 | 3.84 ± 6.05 | Hohe Mut Richtung Gaisbergtal, Obergurgl, Ötztal, Tirol |
| | opp2_C | 46.973183 | 12.280487 | 2601 m | 05/10/2017 | 0.77 ± 6.57 | Großbachtal, Osttirol, Grube Nähe Neuer Reichenbergerhütte |
| *paniculata* | pan_E | 47.7028 | 15.59495 | 1880 m | 13/08/2016 | 7.48 ± 7.30 | Mürzzuschlag, Altenberg an der Rax, Schneealpe, 10 km NW Mürzzuschlag |
| | pan_SW | 44.761191 | 6.276853 | 1487 m | 02/07/2017 | 5.87 ± 6.53 | Champoléon, Hautes-Alpes |
| | pan_W | 46.038943 | 7.954566 | 2244 m | 21/09/2016 | 1.33 ± 5.74 | Mattmark, Saas-Almagell, Wallis |
| | pan1_C | 46.8375 | 11.035944 | 2226 m | 23/08/2016 | 3.84 ± 6.05 | Moräne 1656, Rotmoostal, Obergurgl, Ötztal, Tirol |
| | pan2_C | 46.093589 | 11.837305 | 2160 m | 12/08/2016 | 2.9 ± 6.01 | Parco Nazionale Dolomiti Bellunesi, cime Vette Grandi (Belluno), Italy; Dolomiten |
| *rotundifolia* | rot_C | 47.331115 | 11.468531 | 1654 m | 08/09/2016 | 4.4 ± 6.12 | Issjöchl, Weg Richtung Issanger, Nordtirol |
| | rot_W | 46.139303 | 8.1003498 | 1597 m | 17/08/2017 | 3.98 ± 6.55 | Bielti, Zwischbergental, Megaphorbiée de long du sentier en ubac sous le barrage de Fah |

| | | | | | | | |
|---|---|---|---|---|---|---|---|
| | rot1_E | 47.68186 | 15.60724 | 1530 m | 27/07/2016 | 7.48 ± 7.30 | Mürzzuschlag, Altenberg an der Rax, Schneealpe, 10 km NW Mürzzuschlag |
| | rot1_SW | 43.990111 | 7.670417 | 1700 m | 03/08/2016 | 15.04 ± 5.67 | Monte Pietravecchia, Pigna (Imperia), Liguria, Italy; Alpi Liguri |
| | rot2_E | 47.459564 | 13.829148 | 2048 m | 31/08/2016 | 4.5 ± 6.03 | Stoderzinken, BH20160831-6 |
| | rot2_SW | 43.7053 | 6.2635 | 918 m | 03/08/2017 | 12.5 ± 6.38 | Faverges Seythenex, Haute-Savoie, Montagne de Seythenex |
| *rudolphiana* | rud_C | 46.973184 | 12.280488 | 2601 m | 05/10/2017 | 0.77 ± 6.57 | Großbachtal, Osttirol, Grube Nähe Neuer Reichenbergerhütte |
| *sedoides* | sed_C | 46.62725 | 12.72631 | 2300 m | 18/08/2017 | 3.39 ± 6.89 | Belluno, comune di Sappada, footpath to Calvi hut and Sesis Pass |
| *seguieri* | seg_C | 46.821483 | 11.051433 | 2698 m | 12/09/2016 | 3.84 ± 6.05 | Stempelstelle Rotmoostal, Obergurgl, Ötztal, Tirol |
| | seg_W | 46.505389 | 9.328833 | 2150 m | 22/08/2017 | 2.89 ± 6.23 | Sondrio, comune di Madesimo, Passo dello Spluga |
| *squarrosa* | squ_C | 46.371444 | 11.545333 | 2365 m | 23/09/2017 | 2.71 ± 6.86 | Oberholz/Latemar |
| *stellaris* | ste_E | 47.277409 | 14.095974 | 1520 m | 31/08/2016 | 5.21 ± 6.38 | Sölktal, Winkleralm, BH20160831-23 |
| | ste_W | 46.197332 | 7.512257 | 2235 m | 14/09/2016 | 1.33 ± 5.74 | L´Ar du Tsan, Mont-Noble, Wallis |
| | ste1_C | 46.422778 | 10.188333 | 2163 m | 28/08/2016 | 1.53 ± 5.62 | Strada per rifugio Dosdè, Val Dosdè, Valdidentro (Sondrio), Lombardia, Italy |
| | ste2_C | 46.861417 | 11.002639 | 1948 m | 19/08/2016 | 3.84 ± 6.05 | Hangseite Beilstein, oberhalb Brücke, Obergurl, Ötztal, Tirol |
| *tridactylites* | tri_E | 48.189065 | 16.382294 | 196 m | 05/05/2016 | 11.34 ± 7.70 | BK 20160505-1 |
| | tri_SW | 44.536139 | 5.825167 | 846 m | 12/06/2017 | 7.58 ± 6.64 | Veynes, Hautes-Alpes |
| | tri_W | 46.110645 | 7.0103706 | 1050 m | 25/05/2017 | 2.79 ± 6.42 | Les Marécottes, Rock, Rocher vers léntrée du zoo des Marécottes |

**Supplementary Table S2:** Details on Petri dishes and numbers of seeds laid out per population. See Table S1 for specific location of population.

| Population_ Alps | Temperature condition | Seeds laid | No. Petri dish | Total no. seeds germinated | Total no. seeds not germinated |
|---|---|---|---|---|---|
| ads_C | warm | 100 | 4 | 39 | 55 |
| ads_C | cold | 100 | 4 | 38 | 59 |
| ads_SW | warm | 100 | 4 | 0 | 93 |
| ads_SW | cold | 100 | 4 | 1 | 82 |
| aiz_SW | warm | 98 | 4 | 41 | 46 |
| aiz_SW | cold | 100 | 4 | 71 | 17 |
| aiz1_C | warm | 100 | 4 | 83 | 16 |
| aiz1_C | cold | 100 | 4 | 83 | 12 |
| aiz1_E | warm | 100 | 4 | 90 | 9 |
| aiz1_E | cold | 100 | 4 | 99 | 0 |
| aiz2_C | warm | 100 | 4 | 70 | 23 |
| aiz2_C | cold | 100 | 4 | 87 | 10 |
| aiz2_E | warm | 100 | 4 | 63 | 29 |
| aiz2_E | cold | 100 | 4 | 89 | 3 |
| and_C | warm | 100 | 4 | 0 | 96 |
| and_C | cold | 100 | 4 | 0 | 99 |
| and_E | warm | 60 | 3 | 0 | 56 |
| and_E | cold | 60 | 3 | 0 | 59 |
| and_SW | warm | 100 | 4 | 0 | 97 |
| and_SW | cold | 100 | 4 | 0 | 97 |
| asp_C | warm | 100 | 4 | 0 | 99 |
| asp_C | cold | 100 | 4 | 25 | 74 |
| asp1_SW | warm | 100 | 4 | 0 | 92 |
| asp1_SW | cold | 100 | 4 | 19 | 70 |
| asp1_W | warm | 100 | 4 | 0 | 80 |
| asp1_W | cold | 100 | 4 | 0 | 69 |
| asp2_SW | warm | 100 | 4 | 0 | 97 |
| asp2_SW | cold | 100 | 4 | 0 | 99 |
| asp2_W | warm | 100 | 4 | 0 | 98 |
| asp2_W | cold | 100 | 4 | 0 | 95 |
| asp3_W | warm | 100 | 4 | 0 | 98 |
| asp3_W | cold | 100 | 4 | 0 | 100 |
| bif_C | warm | 99 | 4 | 11 | 79 |
| bif_C | cold | 100 | 4 | 35 | 62 |
| bif_SW | warm | 99 | 4 | 1 | 85 |
| bif_SW | cold | 100 | 4 | 0 | 76 |
| bif_W | warm | 98 | 4 | 1 | 82 |
| bif_W | cold | 98 | 4 | 0 | 84 |
| ble_E | warm | 0 | 1 | 0 | NA |
| ble_E | cold | 0 | 1 | 0 | NA |

| | | | | | |
|---|---|---|---|---|---|
| bry_E | warm | 99 | 4 | 0 | 85 |
| bry_E | cold | 100 | 4 | 0 | 85 |
| bry_SW | warm | 101 | 4 | 0 | 98 |
| bry_SW | cold | 100 | 4 | 0 | 93 |
| bry_W | warm | 99 | 4 | 1 | 92 |
| bry_W | cold | 100 | 4 | 0 | 87 |
| bry1_C | warm | 100 | 4 | 0 | 93 |
| bry1_C | cold | 100 | 4 | 0 | 90 |
| bry2_C | warm | 99 | 4 | 0 | 75 |
| bry2_C | cold | 99 | 4 | 2 | 67 |
| bur_E | warm | 99 | 4 | 0 | 96 |
| bur_E | cold | 100 | 4 | 0 | 100 |
| cae_SW | warm | 100 | 4 | 5 | 91 |
| cae_SW | cold | 100 | 4 | 86 | 12 |
| cae_W | warm | 100 | 4 | 16 | 75 |
| cae_W | cold | 100 | 4 | 40 | 58 |
| cae1_C | warm | 100 | 4 | 14 | 82 |
| cae1_C | cold | 100 | 4 | 28 | 67 |
| cae1_E | warm | 100 | 4 | 20 | 63 |
| cae1_E | cold | 99 | 4 | 70 | 16 |
| cae2_C | warm | 100 | 4 | 13 | 78 |
| cae2_C | cold | 100 | 4 | 49 | 48 |
| cae2_E | warm | 100 | 4 | 25 | 59 |
| cae2_E | cold | 100 | 4 | 88 | 5 |
| cer_W | warm | 0 | 1 | 0 | NA |
| cer_W | cold | 0 | 1 | 0 | NA |
| coc_SW | warm | 100 | 4 | 0 | 47 |
| coc_SW | cold | 100 | 4 | 31 | 12 |
| cot1_W | warm | 100 | 4 | 0 | 94 |
| cot1_W | cold | 100 | 4 | 50 | 39 |
| cot2_W | warm | 100 | 4 | 0 | 96 |
| cot2_W | cold | 100 | 4 | 65 | 31 |
| exa_C | warm | 100 | 4 | 0 | 100 |
| exa_C | cold | 100 | 4 | 0 | 99 |
| exa_W | warm | 100 | 4 | 0 | 100 |
| exa_W | cold | 100 | 4 | 5 | 94 |
| exa1_SW | warm | 100 | 4 | 0 | 93 |
| exa1_SW | cold | 100 | 4 | 71 | 16 |
| exa2_SW | warm | 100 | 4 | 0 | 97 |
| exa2_SW | cold | 100 | 4 | 0 | 100 |
| hos_E | warm | 75 | 3 | 0 | 69 |
| hos_E | cold | 75 | 3 | 22 | 49 |
| hos1_C | warm | 100 | 4 | 0 | 100 |
| hos1_C | cold | 100 | 4 | 93 | 7 |

| | | | | | |
|---|---|---|---|---|---|
| hos2_C | warm | 75 | 3 | 0 | 65 |
| hos2_C | cold | 75 | 3 | 48 | 14 |
| mos_SW | warm | 100 | 4 | 2 | 97 |
| mos_SW | cold | 100 | 4 | 92 | 7 |
| mos1_C | warm | 100 | 4 | 5 | 91 |
| mos1_C | cold | 100 | 4 | 27 | 69 |
| mos1_E | warm | 100 | 4 | 46 | 53 |
| mos1_E | cold | 100 | 4 | 69 | 29 |
| mos2_C | warm | 100 | 4 | 0 | 99 |
| mos2_C | cold | 100 | 4 | 17 | 80 |
| mos2_E | warm | 100 | 4 | 22 | 70 |
| mos2_E | cold | 100 | 4 | 73 | 24 |
| mut_C | warm | 100 | 4 | 38 | 41 |
| mut_C | cold | 100 | 4 | 80 | 10 |
| mut_W | warm | 100 | 4 | 59 | 28 |
| mut_W | cold | 100 | 4 | 98 | 0 |
| opp_E | warm | 100 | 4 | 2 | 93 |
| opp_E | cold | 100 | 4 | 56 | 40 |
| opp_SW | warm | 100 | 4 | 2 | 88 |
| opp_SW | cold | 100 | 4 | 39 | 51 |
| opp_W | warm | 60 | 3 | 0 | 55 |
| opp_W | cold | 58 | 3 | 12 | 40 |
| opp1_C | warm | 100 | 4 | 0 | 92 |
| opp1_C | cold | 100 | 4 | 10 | 86 |
| opp2_C | warm | 100 | 4 | 0 | 96 |
| opp2_C | cold | 100 | 4 | 41 | 56 |
| pan_E | warm | 100 | 4 | 1 | 93 |
| pan_E | cold | 100 | 4 | 63 | 34 |
| pan_SW | warm | 100 | 4 | 0 | 93 |
| pan_SW | cold | 101 | 4 | 14 | 81 |
| pan_W | warm | 50 | 2 | 0 | 47 |
| pan_W | cold | 50 | 2 | 1 | 46 |
| pan1_C | warm | 100 | 4 | 0 | 98 |
| pan1_C | cold | 101 | 4 | 2 | 98 |
| pan2_C | warm | 50 | 2 | 0 | 38 |
| pan2_C | cold | 50 | 2 | 8 | 34 |
| rot_C | warm | 100 | 4 | 96 | 1 |
| rot_C | cold | 100 | 4 | 98 | 1 |
| rot_W | warm | 100 | 4 | 49 | 25 |
| rot_W | cold | 100 | 4 | 60 | 8 |
| rot1_E | warm | 100 | 4 | 86 | 6 |
| rot1_E | cold | 100 | 4 | 93 | 4 |
| rot1_SW | warm | 100 | 4 | 85 | 7 |
| rot1_SW | cold | 100 | 4 | 82 | 14 |

| | | | | | |
|---|---|---|---|---|---|
| rot2_E | warm | 100 | 4 | 94 | 1 |
| rot2_E | cold | 100 | 4 | 92 | 2 |
| rot2_SW | warm | 100 | 4 | 79 | 9 |
| rot2_SW | cold | 100 | 4 | 82 | 4 |
| rud_C | warm | 100 | 4 | 80 | 14 |
| rud_C | cold | 100 | 4 | 97 | 0 |
| sed_C | warm | 100 | 4 | 0 | 99 |
| sed_C | cold | 100 | 4 | 0 | 96 |
| seg_C | warm | 100 | 4 | 1 | 96 |
| seg_C | cold | 100 | 4 | 4 | 88 |
| seg_W | warm | 100 | 4 | 1 | 98 |
| seg_W | cold | 100 | 4 | 6 | 91 |
| squ_C | warm | 100 | 4 | 29 | 68 |
| squ_C | cold | 100 | 4 | 96 | 0 |
| ste_E | warm | 100 | 4 | 50 | 31 |
| ste_E | cold | 100 | 4 | 43 | 34 |
| ste1_C | warm | 100 | 4 | 91 | 3 |
| ste1_C | cold | 100 | 4 | 76 | 3 |
| ste2_C | warm | 100 | 4 | 93 | 5 |
| ste2_C | cold | 100 | 4 | 93 | 3 |
| tri_E | warm | 100 | 4 | 0 | 96 |
| tri_E | cold | 100 | 4 | 0 | 100 |
| tri_SW | warm | 100 | 4 | 0 | 87 |
| tri_SW | cold | 100 | 4 | 0 | 86 |
| tri_W | warm | 100 | 4 | 0 | 96 |
| tri_W | cold | 100 | 4 | 0 | 97 |

**Supplementary Table S3:** Details of Pots and numbers of seeds laid out per population per pot in the common garden experiment. See Table S1 for details on each population.

| Population_ Alps | Seeds laid | Total no. seeds emerged | No. Pots |
|---|---|---|---|
| ads_C | 300 | 14 | 3 |
| ads_SW | 300 | 6 | 3 |
| aiz_SW | 300 | 224 | 3 |
| aiz_W | 300 | 43 | 3 |
| aiz1_C | 300 | 212 | 3 |
| aiz1_E | 300 | 182 | 3 |
| aiz2_C | 300 | 157 | 3 |
| aiz2_E | 300 | 171 | 3 |
| and_C | 300 | 47 | 3 |
| and_E | 300 | 2 | 3 |
| and_SW | 300 | 26 | 3 |
| asp_C | 300 | 132 | 3 |
| asp1_SW | 300 | 148 | 3 |
| asp1_W | 300 | 32 | 3 |
| asp2_SW | 300 | 115 | 3 |
| asp2_W | 300 | 166 | 3 |
| asp3_W | 300 | 183 | 3 |
| bif_C | 300 | 195 | 3 |
| bif_SW | 300 | 127 | 3 |
| bif_W | 300 | 195 | 3 |
| ble_E | 200 | 89 | 2 |
| bry_E | 300 | 131 | 3 |
| bry_SW | 300 | 183 | 3 |
| bry_W | 300 | 138 | 3 |
| bry1_C | 300 | 147 | 3 |
| bry2_C | 300 | 169 | 3 |
| bur_E | 300 | 156 | 3 |
| cae_SW | 300 | 192 | 3 |
| cae_W | 300 | 57 | 3 |
| cae1_C | 300 | 141 | 3 |
| cae1_E | 300 | 117 | 3 |
| cae2_C | 300 | 170 | 3 |
| cae2_E | 300 | 126 | 3 |
| cer_W | 300 | 134 | 3 |
| coc_SW | 300 | 59 | 3 |
| cot1_W | 300 | 133 | 3 |
| cot2_W | 300 | 210 | 3 |
| exa_C | 300 | 204 | 3 |
| exa_W | 300 | 125 | 3 |
| exa1_SW | 300 | 51 | 3 |
| exa2_SW | 300 | 32 | 3 |
| hos_E | 300 | 224 | 3 |
| hos1_C | 300 | 236 | 3 |
| hos2_C | 300 | 173 | 3 |
| mos_SW | 300 | 115 | 3 |
| mos1_C | 300 | 179 | 3 |

| | | | |
|---|---|---|---|
| mos1_E | 300 | 94 | 3 |
| mos2_C | 300 | 91 | 3 |
| mos2_E | 300 | 158 | 3 |
| mut_C | 300 | 195 | 3 |
| mut_W | 300 | 209 | 3 |
| opp_E | 300 | 164 | 3 |
| opp_SW | 300 | 134 | 3 |
| opp_W | 300 | 161 | 3 |
| opp1_C | 300 | 115 | 3 |
| opp2_C | 300 | 223 | 3 |
| pan_E | 300 | 205 | 3 |
| pan_SW | 300 | 99 | 3 |
| pan_W | 300 | 203 | 3 |
| pan1_C | 300 | 219 | 3 |
| pan2_C | 300 | 94 | 3 |
| rot_C | 300 | 146 | 3 |
| rot_W | 300 | 114 | 3 |
| rot1_E | 300 | 128 | 3 |
| rot1_SW | 300 | 93 | 3 |
| rot2_E | 300 | 123 | 3 |
| rot2_SW | 300 | 122 | 3 |
| rud_C | 300 | 160 | 3 |
| sed_C | 300 | 4 | 3 |
| seg_C | 300 | 81 | 3 |
| seg_W | 300 | 36 | 3 |
| squ_C | 300 | 167 | 3 |
| ste_E | 300 | 9 | 3 |
| ste_W | 300 | 5 | 3 |
| ste1_C | 300 | 38 | 3 |
| ste2_C | 300 | 118 | 3 |
| tri_E | 300 | 9 | 3 |
| tri_SW | 300 | 1 | 3 |
| tri_W | 300 | 3 | 3 |

**Supplementary Table S4:** Post-hoc results (bonferroni method) of the generalized linear mixed model for emergence, listing life forms (c = herbaceous chamaeophyte, h = hemicryptophyte, t = therophyte).

| Distribution | comparison | Est. (%) | SE | z.ratio | p value |
|---|---|---|---|---|---|
| wide | c-h | -0.0255 | 0.0778 | -0.327 | 1.000 |
| wide | c-t | 0.2311 | 0.0283 | 8.151 | <0.001 |
| wide | h-t | 0.2565 | 0.0771 | 3.329 | 0.003 |
| narrow | c-h | -0.0239 | 0.0718 | -0.332 | 1.000 |
| narrow | c-t | 0.2087 | 0.0472 | 4.423 | <0.001 |
| narrow | h-t | 0.2326 | 0.0648 | 3.589 | 0.001 |

Confidence level 0.95; Conf-level and p value adjustment: bonferroni method for 3 estimates/tests

**Supplementary Table S5:** Results of random effects of the mixed models, used to analyze response variables (A-F). The variance components of the full models are used to calculate ICC (Suppl. Fig. S4).

| **(A) Germination percentage** | | |
|---|---|---|
| Random effects | variance components | |
| Species | 5.642 | 3.136 |
| Population | 1.364 | 1.359 |
| Residuals | pi^2/3 ° | pi^2/3 ° |
| Fixed factors | - | 2.277 |
| $R^2$ | 22.63% | 67.30% |
| **(B) MTG** | | |
| Random effects | variance components | |
| Species | 47.119 | 4.900 |
| Population | 45.470 | 48.465 |
| Residuals | 24.917 | 19.627 |
| Fixed factors | - | 53.103 |
| $R^2$ | 42.11% | 84.43% |
| **(C) Emergence percentage** | | |
| Random effects | variance components | |
| Species | 1.069 | 0.234 |
| Population | 0.300 | 0.306 |
| Residuals | pi^2/3 ° | pi^2/3 ° |
| Fixed factors | - | 0.641 |
| $R^2$ | 0.54% | 26.43% |
| **(D) MTE** | | |
| Random effects | variance components | |
| Species | 0.524 | 0.171 |
| Population | 0.422 | 0.436 |
| Residuals | 0.505 | 0.505 |
| Fixed factors | - | 0.343 |
| $R^2$ | 23.56% | 65.29% |
| **(E) Survival percentage ‘gs’** | | |
| Random effects | variance components | |
| Species | 0.809 | 0.737 |
| Population | 3.086 | 0.393 |
| Residuals | pi^2/3 ° | pi^2/3 ° |
| Fixed factors | - | 1.776 |
| $R^2$ | 28.66% | 46.90% |
| **(F) Survival percentage ‘ow’** | | |
| Random effects | variance components | |
| Species | 0.097 | 0.000 |
| Population | 0.012 | 0.011 |
| Residuals | pi^2/3 ° | pi^2/3 ° |
| Fixed factors | - | 0.080 |
| $R^2$ | 2.38% | 2.69% |

$R^2$ = variance explained by null model (m0) or full model (m1); - = not applicable,

° Nakagawa and Schielzeth (2010, 2013; for models without overdispersion)

**Supplementary Table S6:** Results of linear regressions on the coefficients of variation (CV) at species level, comparing intraspecific variation between widespread and narrow distributed *Saxifraga* spp. The last row provides results of a linear regression testing the CV of average annual temperatures (Tave) from seed collection sites.

| Variable | Comparison | Est. (SE) | Df | Sum Sq | Mean Sq | F | p value |
|---|---|---|---|---|---|---|---|
| Germination (%) (15/5°C) | Distribution wide-narrow | -0.0003 (± 0.04) | 1 | 0.00 | 0.00 | 0.009 | 0.928 |
| | Residuals | 0 (± 0.10) | 12 | 1.21 | 0.10 | | |
| MTG (days) (15/5°C) | Distribution wide-narrow | -0.0008 (± 0.00) | 1 | 0.002 | 0.002 | 0.758 | 0.403 |
| | Residuals | 0.05 (± 0.002) | 5 | 0.026 | 0.002 | | |
| Emergence (%) | Distribution wide-narrow | 0.00002 (± 0.01) | 1 | 0.000 | 0.000 | 0.001 | 0.971 |
| | Residuals | 0.00 (± 0.05) | 16 | 0.813 | 0.051 | | |
| MTE (recording date) | Distribution wide-narrow | -0.008 (± 0.001) | 1 | 0.02 | 0.02 | 6.902 | 0.020* |
| | Residuals | 0.00 (± 0.003) | 14 | 0.04 | 0.00 | | |
| Annual Tave (°C) | Distribution wide-narrow | -0.13 (± 0.02) | 1 | 0.45 | 0.45 | 7.091 | 0.017* |
| | Residuals | 0 (± 0.06) | 16 | 1.02 | 0.06 | | |

Est. (SE) = Estimate and Standard Error, Df = degrees of freedom, Sum Sq = sum of squares, Mean sq = Mean sum of squares, * = significant on $\alpha<0.05$

**Supplementary Table S7:** Spearman correlation coefficients (R) of germination and emergence (data from 2017 and 2018), in warm and cold temperature, and emergence and survival 'gs' (data from 2017), for each distribution

| Variables | | Distribution | R (spearman) | p value |
|---|---|---|---|---|
| Germination warm | Emergence | wide | 0.099 | 0.747 |
| Germination warm | Emergence | narrow | 0.280 | 0.433 |
| Germination cold | Emergence | wide | 0.203 | 0.505 |
| Germination cold | Emergence | narrow | 0.718 | 0.019 |
| Emergence | Survival 'gs' | wide | 0.685 | 0.014 |
| Emergence | Survival 'gs' | narrow | 0.500 | 0.391 |

## 2 Supplementary Text

General information on model building

We started with intercept models that included only random effects (null model, m0), i.e., species and population nested within species. We then included the fixed factors and in case of germination and MTG, the interaction effect. Significance of fixed factors were tested via likelihood ratio tests; contrast coding was applied when the interaction term was significant, to avoid conflicts introduced by interaction terms in the model matrix (Levy, 2014). A significance level of 5% was chosen for all models, and model assumptions (normality and heteroscedasticity of residuals) were checked via residual diagnostic plots ('ggResidpanels'; Goode and Rey, 2019), which were met. In both types of models, i.e., generalized and linear mixed models, variance estimation was done via Maximum likelihood-method.

To determine the variance explained by fixed effects in the models, a $R^2$ for the null model ($R^2$m0) and the conditional (full) model ($R^2$m1), respectively, were calculated, as suggested by Nakagawa and Schielzeth, (2013). Furthermore, we extracted the variance components of the models (i.e., random factors) to determine the intraclass correlation coefficient (icc; variance of random terms divided by variance of random term plus residual variance; Nakagawa et al., (2017).

Intraclass correlation coefficient (ICC)

In an anova-like framework, the variance explained by the model terms can be expressed by the intraclass correlation coefficient (ICC). The ICC is calculated by dividing the random effect variance, (σ2i), by the total variance, i.e., the sum of the random effect variances and the residual variance (σ2ε). Residual variance in a generalized linear mixed model with binomial structure without overdispersion is assumed as pi^2/3 (Nakagawa and Schielzeth, 2010, 2013). As the models were not over dispersed, adding a multiplicative overdispersion term to the residual variance was not necessary to calculate the ICC.

Goode, K., and Rey, K. (2019). ggResidpanel: Panels and Interactive Versions of Diagnostic Plots using "ggplot2." Packag. R Softw. Stat. Comput. CRAN Repos. Available at: https://cran.r-project.org/package=ggResidpanel [Accessed November 15, 2021].

Levy, R. (2014). Using R formulae to test for main effects in the presence of higher-order interactions. arxiv.org/abs/1405.2094.

Nakagawa, S., Johnson, P. C. D., and Schielzeth, H. (2017). The coefficient of determination R2 and intra-class correlation coefficient from generalized linear mixed-effects models revisited and expanded. J. R. Soc. Interface 14. doi:10.1098/rsif.2017.0213.

Nakagawa, S., and Schielzeth, H. (2010). Repeatability for Gaussian and non-Gaussian data: A practical guide for biologists. Biol. Rev. 85, 935–956. doi:10.1111/j.1469-185X.2010.00141.x.

Nakagawa, S., and Schielzeth, H. (2013). A general and simple method for obtaining R2 from generalized linear mixed-effects models. Methods Ecol. Evol. 4, 133–142. doi:10.1111/j.2041-210x.2012.00261.x.

## 3 Supplementary Figures

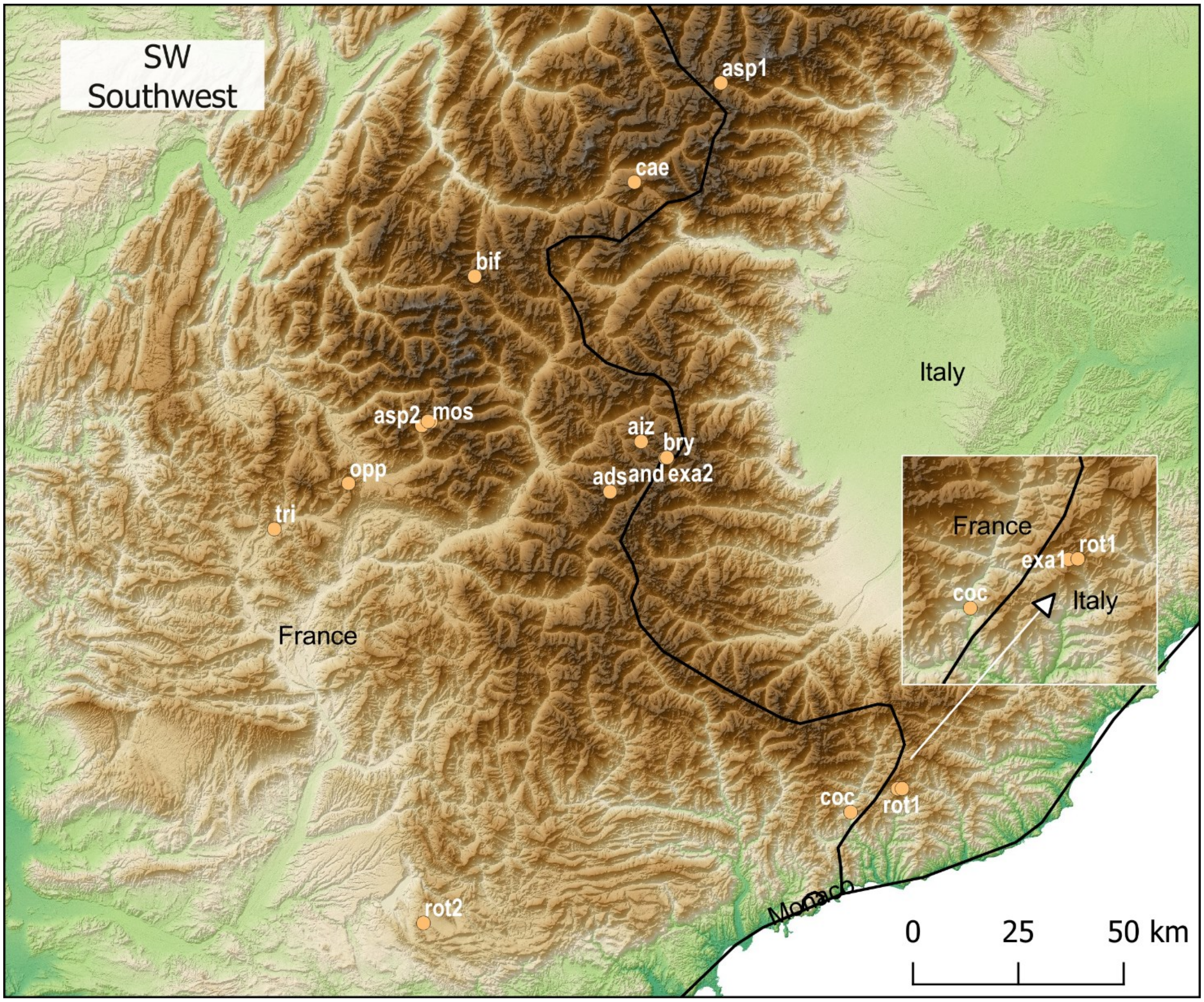

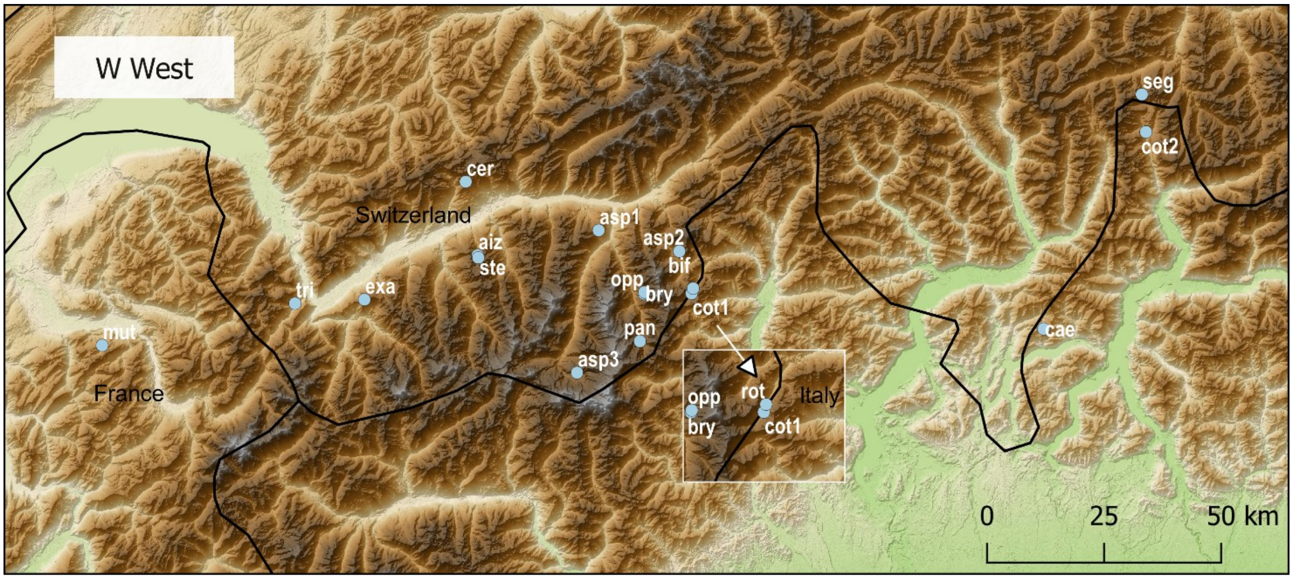
W West
seg
cot2
cer
Switzerland
asp1
asp2
aiz
ste
bif
opp
bry
tri
exa
cot1
mut
pan
cae
asp3
France
rot
Italy
opp
bry
cot1
0
25
50 km

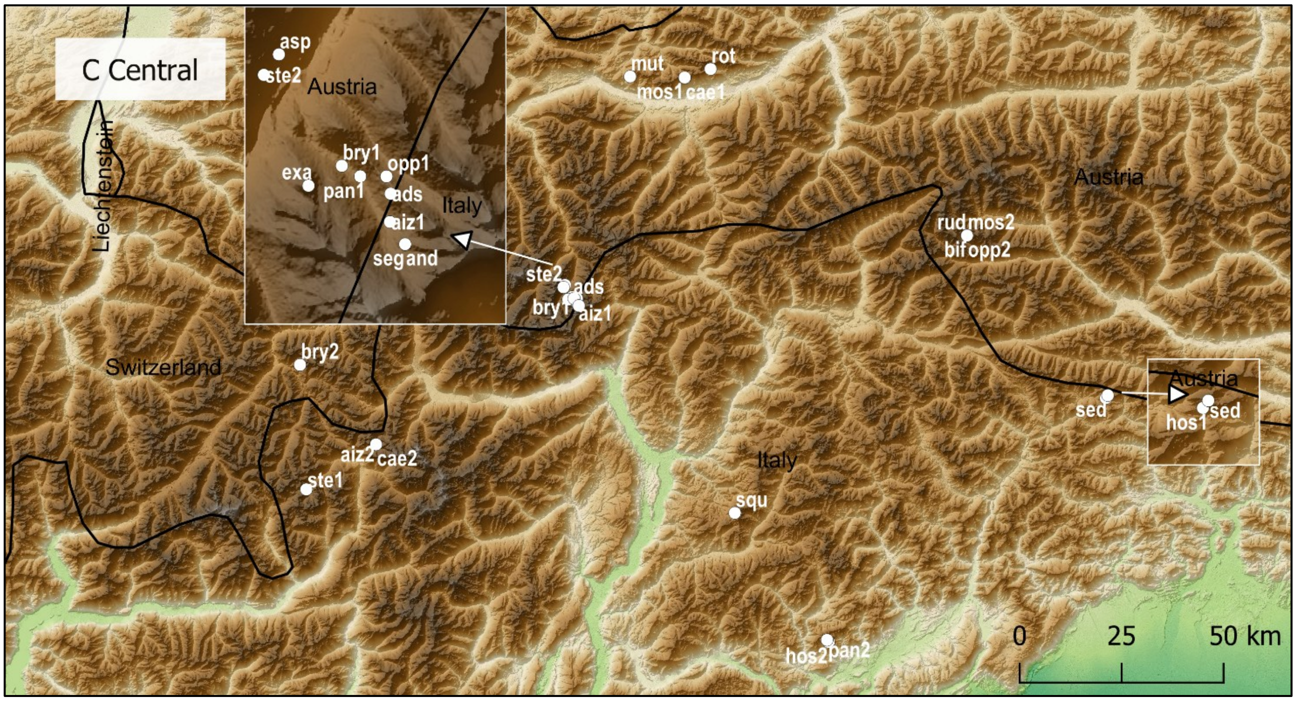
C Central
asp
ste2
Austria
mut
rot
mos1
cae1
Liechtenstein
bry1
opp1
exa
pan1
ads
Italy
aiz1
seg
and
Austria
rud
mos2
bif
opp2
ste2
ads
bry1
aiz1
bry2
Switzerland
Austria
sed
sed
hos1
aiz2
cae2
ste1
Italy
squ
hos2
pan2
0
25
50 km

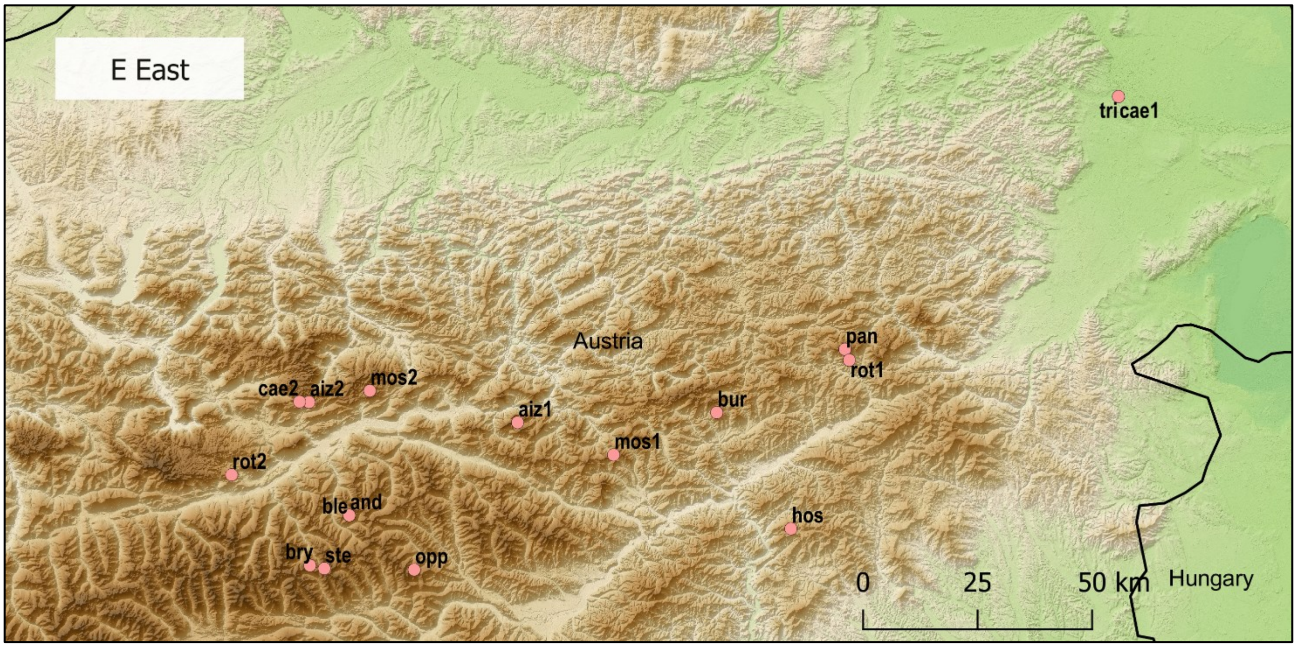


**Supplementary Figure S1.** Map details for each collecting point: See location details in Table S1 for each population. Maps were produced using QGIS Software.

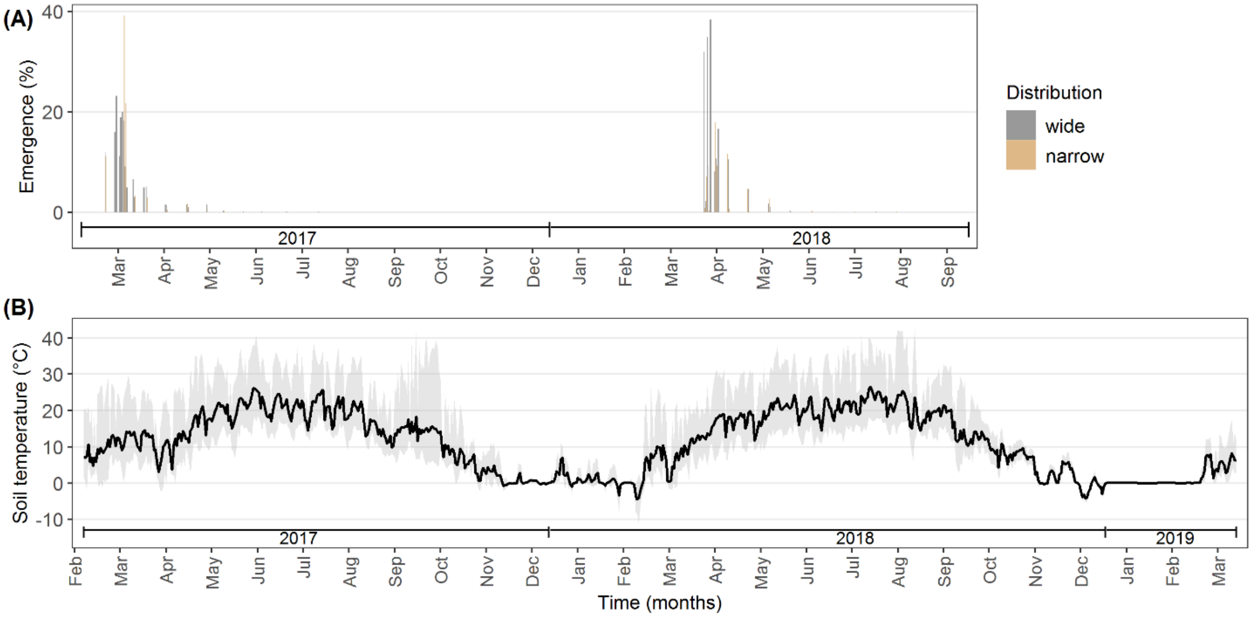


**Supplementary Figure S2**: (A) Seedling emergence of *Saxifraga* spp. in the common garden throughout the duration of the experiment grouped in distribution (wide, narrow). (B) Soil temperature (2 cm depth) measured in the common garden during the experiment (years 2017-2019). Black line denotes average daily temperature, grey ribbon illustrates daily minima and maxima temperatures.

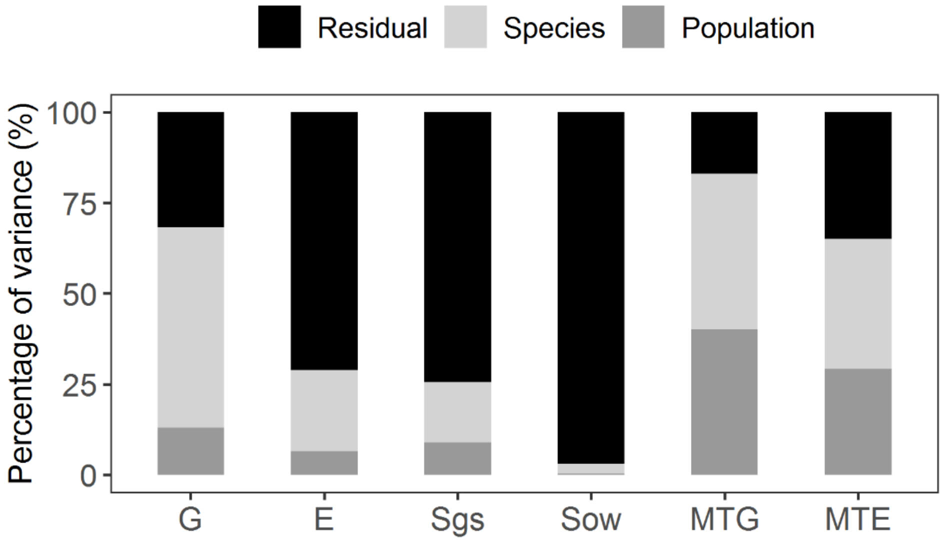


**Supplementary Figure S3**: Variance decomposition at the species and the population level from generalized- and linear- mixed models, for germination percentage (G), emergence percentage (E) survival percentage ‘gs’ (Sgs), survival percentage ‘ow’ (Sow), MTG, and MTE. Residual variance is the variance that remains unexplained.
